# The identification of distinct protective and susceptibility mechanisms for hip osteoarthritis: findings from a genome-wide association study meta-analysis of minimum joint space width and Mendelian randomisation cluster analyses

**DOI:** 10.1016/j.ebiom.2023.104759

**Published:** 2023-08-22

**Authors:** Benjamin G. Faber, Monika Frysz, Cindy G. Boer, Daniel S. Evans, Raja Ebsim, Kaitlyn A. Flynn, Mischa Lundberg, Lorraine Southam, April Hartley, Fiona R. Saunders, Claudia Lindner, Jennifer S. Gregory, Richard M. Aspden, Nancy E. Lane, Nicholas C. Harvey, David M. Evans, Eleftheria Zeggini, George Davey Smith, Timothy Cootes, Joyce Van Meurs, John P. Kemp, Jonathan H. Tobias

**Affiliations:** a Musculoskeletal Research Unit, University of Bristol, UK; b Medical Research Council Integrative Epidemiology Unit at the University of Bristol, UK; c Department of Internal Medicine, Erasmus Medical Centre, Rotterdam, the Netherlands; d California Pacific Medical Center Research Institute, San Francisco, USA; e Division of Informatics, Imaging and Data Sciences, The University of Manchester, UK; f Mater Research Institute, The University of Queensland, Woolloongabba, Australia; g Institute for Molecular Bioscience, The University of Queensland, St Lucia, Australia; h UQ Frazer Institute, The University of Queensland, Woolloongabba, Australia; i Institute of Translational Genomics, Helmholtz Zentrum München – German Research Centre for Environmental Health, Neuherberg, Germany; j Centre for Arthritis and Musculoskeletal Health, University of Aberdeen, UK; k Center for Musculoskeletal Health, University of California Davis, Sacramento, USA; l Medical Research Council Lifecourse Epidemiology Centre, University of Southampton, UK; m NIHR Southampton Biomedical Research Centre, University of Southampton and University Hospital Southampton NHS Foundation Trust, UK; n Technical University of Munich and Klinikum Rechts der Isar, TUM School of Medicine, Germany

**Keywords:** Osteoarthritis, Genome-wide association study, Mendelian randomisation, Cartilage

## Abstract

**Background:**

Hip minimum joint space width (mJSW) provides a proxy for cartilage thickness. This study aimed to conduct a genome-wide association study (GWAS) of mJSW to (i) identify new genetic determinants of mJSW and (ii) identify which mJSW loci convey hip osteoarthritis (HOA) risk and would therefore be of therapeutic interest.

**Methods:**

GWAS meta-analysis of hip mJSW derived from plain X-rays and DXA was performed, stratified by sex and adjusted for age and ancestry principal components. Mendelian randomisation (MR) and cluster analyses were used to examine causal effect of mJSW on HOA.

**Findings:**

50,745 individuals were included in the meta-analysis. 42 SNPs, which mapped to 39 loci, were identified. Mendelian randomisation (MR) revealed little evidence of a causal effect of mJSW on HOA (${OR}_{IVW}$ 0.98 [95% CI 0.82–1.18]). However, MR-Clust analysis suggested the null MR estimates reflected the net effect of two distinct causal mechanisms cancelling each other out, one of which was protective, whereas the other increased HOA susceptibility. For the latter mechanism, all loci were positively associated with height, suggesting mechanisms leading to greater height and mJSW increase the risk of HOA in later life.

**Interpretations:**

One group of mJSW loci reduce HOA risk via increased mJSW, suggesting possible utility as targets for chondroprotective therapies. The second group of mJSW loci increased HOA risk, despite increasing mJSW, but were also positively related to height, suggesting they contribute to HOA risk via a growth-related mechanism.

**Funding:**

Primarily funded by the 10.13039/501100000265Medical Research Council and 10.13039/100010269Wellcome Trust.

**Research in context**

**Evidence before this study**

Osteoarthritis is the most common cause of hip pain worldwide. One previous study found 4 genetic loci associated with cartilage thickness at the hip, some of which showed nominal associations with hip osteoarthritis suggesting that a portion of hip osteoarthritis (HOA) heritability is conveyed through cartilage thickness.

**Added value of this study**

This study presents a genome-wide association meta-analysis of minimum joint space width (mJSW), a proxy of cartilage thickness. It identified 39 loci that contain mJSW associated single nucleotide polymorphisms (SNPs). Interestingly, initial Mendelian randomisation (MR) results showed no causal effect of decreasing mJSW on hip osteoarthritis risk. Using MR clustering analyses, 3 groups of mJSW loci were revealed based on associations with HOA risk: Cluster one was associated with larger mJSW and lower HOA risk; Cluster two was associated with larger mJSW, increased HOA risk and increased height; Cluster three was unrelated to HOA risk. The equivalent opposing effects of Cluster one and two loci explained the initial null MR results. Subsequent, fine mapping techniques revealed the likely causal genes implicated by these genetic associations.

**Implications of all the available evidence**

The evidence suggests mJSW associated loci can affect HOA risk in two distinct clusters; those that decrease HOA risk with increasing mJSW and those that increase HOA risk via increasing mJSW. The first group of SNPs most likely act via cartilage mediated pathways, suggesting possible utility as targets for chondroprotective therapeutics. In contrast, the latter group of SNPs are associated with greater height and likely act through growth-related mechanisms which might have less therapeutic utility.

## Introduction

Hip osteoarthritis (HOA) is the commonest cause of pain and loss of function of the hip worldwide.^1^ It is a disease of the whole joint with multiple biological pathways implicated in its pathogenesis, involving cartilage, bone and synovium.^2^ The prevalence of HOA is approximately 10% and is predicted to increase.^1^^,^^3^ Currently there are no known drugs to prevent disease and/or symptomatic progression, leaving total hip replacement (THR) as the treatment of choice for those with end-stage disease. As a result, HOA costs European countries over €400 billion/year in both direct and indirect healthcare costs illustrating its substantial health and economic burden.^4^ A better understanding of the pathogenesis of HOA may uncover new opportunities for treatment, prevention and early diagnosis.

A key component of HOA pathogenesis is the loss of cartilage, and this is often seen as a narrowing of the joint space on imaging.^5^ A standardised measure of joint space is minimum joint space width (mJSW), which serves as a proxy of cartilage thickness in large epidemiological studies.^5^^,^^6^ A well-powered systematic review found hip mJSW to have little association with hip pain which counters the idea of it being a useful predictor of disease.^7^ One reason for this could be the heterogeneity of pathways that affect cartilage thickness. For example, a tall individual has a higher risk of HOA^8^ and would be expected to have a wider joint space. Whereas other individuals might have altered cartilage metabolism and homeostasis that predisposes them to early cartilage loss, a smaller mJSW and with this an increased HOA risk.^9^ These opposing disease pathways are difficult to contextualise and understand using conventional epidemiological approaches.

Genome-wide association studies (GWAS) offer the opportunity to identify genes and their biological pathways that predispose an individual to disease and which might offer potential therapeutic targets.^10^^,^^11^ To date, 45 independent genetic loci have been associated with HOA, but the underlying genetic pathways causing disease remain largely unclear.^12^ A more focused GWAS of mJSW might help to identify pathways involved in cartilage metabolism that would be seen as a priority for therapeutic development.^13^ In addition, post-GWAS methods such as Mendelian randomisation (MR) can test if observed associations are causal, rather than being confounded, and using newer techniques cluster genetic loci into effect groups,^14^^,^^15^ potentially discovering previously unseen and unknown opposing genetic effects.^16^

An earlier GWAS found four independent loci associated with mJSW obtained from antero-posterior (AP) radiographs, many of which showed nominal associations with HOA.^17^ Larger sample sizes and updated genotype reference panels provide the opportunity for a more comprehensive characterization of mJSW genetic architecture. The UK Biobank study (UKB) has recently conducted over 40,000 high-resolution dual-energy X-ray absorptiometry (DXA) scans of the hip that have been automatically annotated for mJSW.^3^ The present study aimed to conduct a GWAS meta-analysis of hip mJSW combining X-ray and DXA cohorts to maximize the study power, and then explore the genetic architecture of mJSW and its relationship with HOA. Subsequently, we aimed to evaluate causal effects of mJSW on HOA risk using MR and cluster analyses, to allow for the possibility that distinct sets of SNPs associated with mJSW map to directionally opposite causal pathways.^15^

## Methods

### Cohort descriptions

GWAS cohorts comprised the UKB, The Rotterdam Study (RS) I&II, Osteoporotic Fractures in Men (MrOS) Study and Study of Osteoporotic Fractures (SOF). mJSW was measured automatically in UKB and manually in RS, MrOS and SOF (see Supplementary Methods).

### Genome-wide association study

GWAS for mJSW were conducted separately in UKB, RS I&II, SOF and MrOS. In each cohort, mJSW was stratified by sex and adjusted for age, ancestry principal components, and in addition study site in the case of MrOS and SOF. Given potential relationships between mJSW and height, a further GWAS was performed including height adjustment for each cohort. Residuals resulting from female and male analysis were standardised to mean = 0, SD = 1, and then combined into a single outcome for GWAS. UKB used a linear mixed model for GWAS, implemented in BOLT-LMM (v2.3),^18^ SOF, MrOS used an OLS linear regression model implemented in PLINK^19^ and RS1 and RS2 used RVtests.^5^ RS I&II were imputed to Haplotype Reference Consortium (HRC v.1.1), UK Biobank release V3 was imputed to 3 reference panels (UK10K, 1000 Genomes and HRC) and SOF and MrOS were imputed to 1000 Genomes. All cohorts used the hg19 build.

### Statistics and meta-analysis

Before meta-analysis, quality control of summary statistics was performed using EasyQC.^20^ Briefly, missing data, mono-allelic SNVs, implausible values (Linear regression: *P* > 1, infinite SE, beta >10, EAF>1.) and duplicates were removed from the data. We excluded variants with poor imputation quality (INFO <0.4) and minor allele frequency ≤0.005. Allele coding was harmonized across cohorts (A/T/C/G or I/D) and allele frequency checked against HRC imputed reference (http://www.haplotype-reference-consortium.org/) to identify possible allele coding errors. P-Z scatter plots were inspected for problems with beta estimates, standard errors and *P* values. Cleaned files were used to perform an inverse variance weighted fixed effects meta-analysis was performed with METAL.^21^ Following the meta-analysis SNPs were only considered if they were in more than one cohort and a SNP heterogeneity below a prespecified threshold (I^2^ ≤ 30). A separate GWAS meta-analysis was conducted excluding UKB so that mJSW_DXA_ and mJSW_X-ray_ could be compared. Genome-wide statistical significance threshold was set at a P-value less than 5 × 10^−8^ (linear regression).

Linkage disequilibrium score regression and genome-wide conditional and joint complex trait analysis (GCTA-COJO).

Linkage disequilibrium (LD) score regression (LDSC) v1.0.1 was used to estimate SNP heritability, and the genetic correlation between mJSW and several other traits, including HOA, height, and body mass index (BMI) (see Supplementary Methods).^12^^,^^22^ In addition, the genetic correlation between mJSW_DXA_ and mJSW_X-ray_ was examined. A European based LD reference panel was used, and analysis was limited to HapMap3 SNPs (therefore excluding major histocompatibility regions).^22^ Conditional and joint analysis (GCTA-COJO) was performed in conjunction with a UKB reference panel to identify statistically independent mJSW associated signals.^23^

### Mendelian randomisation and MR cluster analysis

The conditionally independent mJSW lead SNPs were used as genetic instruments for MR analyses to investigate the causal effect of mJSW on HOA, using the TwoSampleMR package v0.5.6 in R.^24^ The HOA GWAS was a meta-analysis combining the latest genetics of osteoarthritis consortium HOA GWAS without UKB^12^ and an updated UKB HOA GWAS removing those individuals with mJSW measures to avoid sample overlap (see Supplementary Methods). Steiger filtering was applied to demonstrate the exposure instruments were upstream of the outcome. Inverse variance weighted (IVW) analysis was used as the primary method, with MR Egger, weighted median, simple mode and weighted mode approaches as sensitivity analyses.^14^ MR-Clust was applied in relation to HOA to group variants into distinct groups with similar causal estimates.^15^ This method, which may help to identify different causal mechanisms underlying HOA, is used when heterogeneity in causal effect estimates for a complex trait is observed, and different biological mechanisms are suspected. Two sample MR was then used to quantify cluster specific effects on HOA and height. A previous GWAS of height (GWAS ID: ukb-b-10787), available via the IEU open GWAS project,^25^ was used.

### Gene prioritisation and downstream analyses

Initially, the independent mJSW lead SNPs were looked-up in a previous GWAS of height (GWAS ID: ukb-b-10787) and BMI (GWAS ID: ukb-b-19953) in UKB^25^ and HOA. SNPs were prioritised based on MR-Clust results and a look-up in previous height and HOA summary statistics. In these fine mapping analyses that used the coloc R package, we compared 100 kb regions on either side of the lead mJSW SNP in the mJSW and HOA GWAS to look for shared signals.^26^ Then generalised gene-set analysis of GWAS data (MAGMA v1.08)^27^ was implemented in Functional Mapping and annotation of GWAS (FUMA) tool.^28^ Briefly, SNPs were mapped to the protein coding genes using default settings (SNP-wise (mean) model for gene test) and gene-set analysis was performed using 10,894 gene sets obtained from MsigDB v5.2. In addition, the list of mapped genes was annotated for overlapping gene ontology biological processes genes using PANTHER.^29^ Subsequently, the expression quantitative trait loci (eQTL) database GTEx was searched for each leading SNP to identify cis-acting effects, with cultured fibroblasts considered the most relevant tissue. LocusFocus was used to conduct Bayesian colocalisation with all expressed genes over 100 kb either side of the sentinel SNP.^26^^,^^30^^,^^31^ To further identify which cis-genes share the same causal variants, we used colocalisation to look at eQTL data assessed on highly degraded (diseased) and less degraded (healthy) cartilage, and synovial tissue retrieved following knee and hip joint replacements.^32^ When referring to the posterior probability (PP) obtained from colocalisation analyses we are referring to the fourth PP indicating a shared causal signal. We considered a SNP to colocalise with an eQTL if the PP was >80%. In addition, regulatory elements of non-coding human genome were identified using RegulomeDB.^33^

### Ethics

All participants provided informed consent for this study and ethical approval was gained from UK Biobank (application number 17295) which is overseen by the Ethics Advisory Committee and received approval from the National Information Governance Board for Health and Social Care and Northwest Multi-Centre Research Ethics Committee (11/NW/0382).

### Role of funders

None of the funders had any role in study design, data collection, data analyses, interpretation, or the writing of this manuscript.

## Results

### Genome wide association analysis

**Fig. 1:**
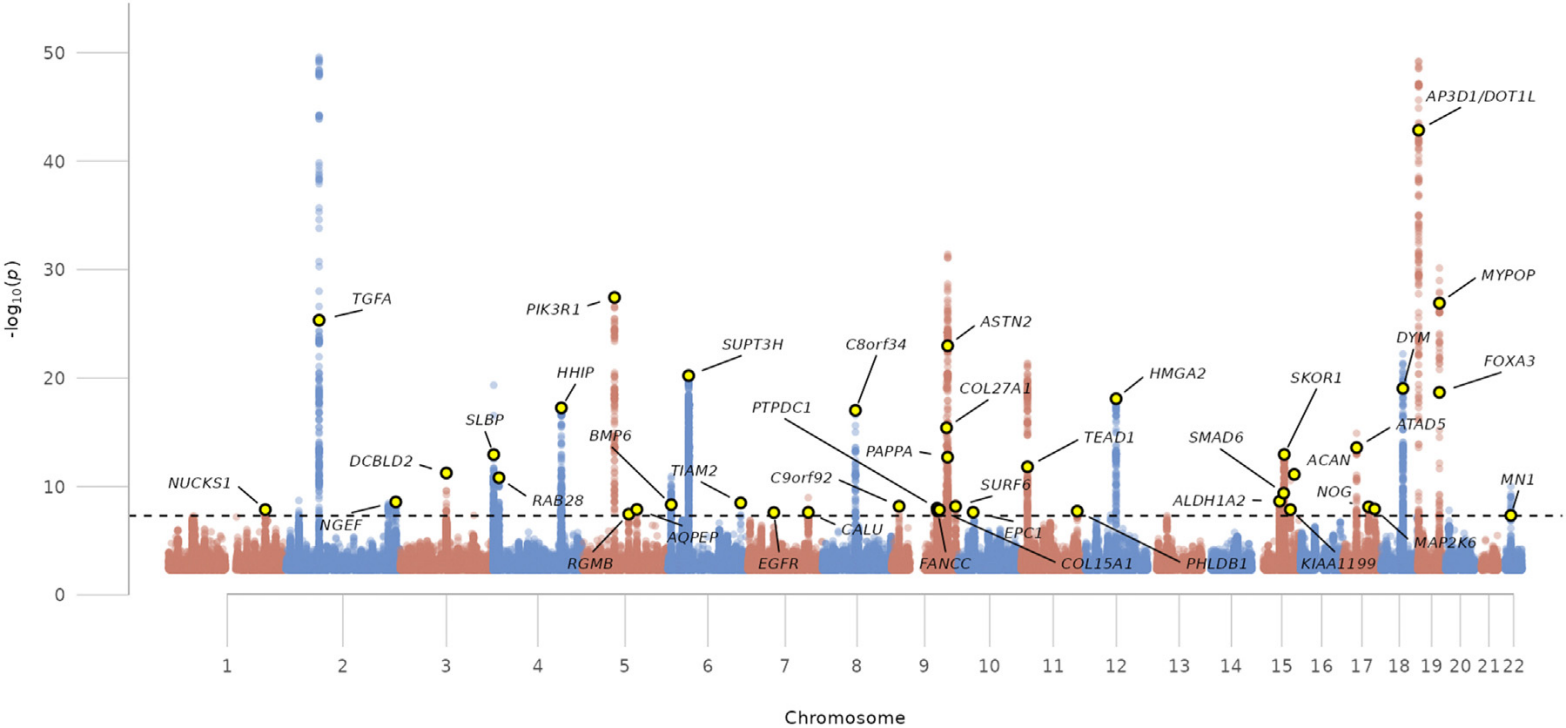
Manhattan plot showing mJSW genome-wide meta-analysis results. The dashed black line denotes the threshold for declaring genome-wide significance (Linear regression: *P* ≤ 5.0 × 10^−8^). Yellow circles represent not previously reported mJSW loci (defined as > 1 MB from previously known genome-wide significant mJSW variants).

We conducted a GWAS meta-analysis of hip mJSW in 50,745 participants from 5 cohorts, of whom 24,429 were males and 26,316 females with a mean age of 65.1 years (range 45–97 years), height of 169.7 cm (135–204 cm), weight of 75.5 kg (34–171 kg) and mJSW of 3.05 mm (0.0–7.4 mm) (Supplementary Table S1). Following conditional analyses, 42 independent SNPs were identified at genome-wide significance (Linear regression: *P* ≤ 5·0 × 10^−8^) (Supplementary Fig. S1.1–S1.42), together accounting for 4.6% of mJSW variance (Table 1, Fig. 1).^17^ The identified SNPs mapped to 39 loci, of which 35 had not previously been associated with mJSW (defined as >1 MB from previously reported variants^17^). mJSW SNP heritability (h^2^) was 0.20 (95% CI 0.16, 0.25), and there was moderate genomic inflation (λ = 1.11; UKB λ = 1.10, MrOS 1.02, SOF 1.00, RS1 1.01, RS2 1.00). However, the intercept from LDSC, and the ratio attenuation statistic (Intercept = 1.01 [Standard error = 0.01]/RPS = 0.28 [0.15]) suggested that most of the inflation reflected polygenicity rather than confounding due to population stratification or relatedness (Supplementary Fig. S2). Equivalent results were obtained in a further GWAS following height adjustment (Supplementary Fig. S3).

**Table 1:** Conditionally independent minimum joint space width single nucleotide polymorphisms, and their associations with height, BMI, and HOA risk.

RSID	CHR	BP	C.GENE	EA	NEA	EAF	Cluster	Cluster prob	mJSW beta	mJSW *P*	HOA Beta	HOA *P*	Height Beta	Height *P*	BMI Beta	BMI *P*
rs7571789	2	70,714,793	*TGFA*	C	T	0.48	1	1	0.09	2.62 × 10^−50^	−0.06	5.32 × 10^−18^	0.00	0.11	−0.01	3.00 × 10^−03^
rs2236996	4	1,703,646	*SLBP*	A	G	0.48	1	0.99	0.05	1.15 × 10^−13^	−0.03	6.74 × 10^−04^	−0.01	6.00 × 10^−07^	0.00	0.11
rs10948155	6	44,687,957	*SUPT3H*	T	C	0.65	1	0.99	0.06	6.07 × 10^−21^	−0.05	9.17 × 10^−11^	0.00	0.78	0.01	1.70 × 10^−03^
rs35199713	6	155,415,593	*TIAM2*	G	A	0.03	1	0.82	0.11	3.25 × 10^−09^	−0.05	0.03	0.01	0.02	−0.02	6.50 10^−03^
rs17172430	7	55,122,650	*EGFR*	A	G	0.12	1	0.56	0.05	2.51 × 10^−08^	−0.02	0.1	0.00	0.93	0.00	0.67
rs7846438	8	69,578,824	*C8orf34*	A	G	0.77	1	1	0.06	9.65 × 10^−18^	−0.04	1.48 × 10^−06^	0.00	0.82	0.01	0.006
rs4979342	9	116,905,618	*COL27A1*	C	T	0.27	1	1	0.06	3.88 × 10^−16^	−0.03	9.40 × 10^−05^	0.00	0.24	0.00	0.52
rs76164690	10	32,590,362	*EPC1*	T	G	0.86	1	0.93	0.05	2.37 × 10^−08^	−0.06	1.59 × 10^−07^	−0.01	5.90 × 10^−06^	0.01	9.10 × 10^−03^
rs11857461	15	58,319,690	*ALDH1A2*	C	T	0.49	1	0.92	0.04	2.26 × 10^−09^	−0.02	0.01	0.00	0.47	0.01	9.70 × 10^−03^
rs34656141	19	2,158,228	*AP3D1*	T	C	0.4	1	0.96	0.09	1.42 × 10^−43^	−0.03	2.34 × 10^−05^	0.02	2.00 × 10^−76^	0.00	0.11
rs2106973	22	28,055,460	*MN1*	G	A	0.48	1	0.95	0.03	4.63 × 10^−08^	−0.02	6.95 × 10^−03^	0.01	1.70 × 10^−13^	0.00	0.81
rs981269	4	12,897,698	*RAB28*	T	C	0.77	2	1	0.05	1.55 × 10^−11^	0.05	2.71 × 10^−07^	0.02	5.10 × 10^−30^	0.00	0.46
rs7711053	5	67,822,620	*PIK3R1*	G	A	0.38	2	1	0.07	3.74 × 10^−28^	0.05	1.57 × 10^−12^	0.01	1.90 × 10^−04^	−0.01	4.70 × 10^−03^
rs270417	6	7,729,614	*BMP6*	T	C	0.72	2	0.7	0.04	4.69 × 10^−09^	0.02	0.01	0.03	7.10 × 10^−87^	−0.01	4.40 × 10^−04^
rs7869550	9	119,134,796	*PAPPA*^d^	A	G	0.8	2	1	0.06	2.01 × 10^−13^	0.06	2.15 × 10^−09^	0.03	1.40 × 10^−74^	0.00	0.27
rs76248879	9	119,325,659	*ASTN2*^d^	A	T	0.87	2	1	0.1	1.07 × 10^−23^	0.10	2.18 × 10^−11^	Proxy NA			
rs597974^a^	9	136,144,297	*SURF6*	A	G	0.68	2	0.92	0.04	6.82 × 10^−09^	0.03	5.44 × 10^−03^	0.00	4.1 × 10^−03^	0.00	0.15
rs34651525	11	12,846,729	*TEAD1*	T	A	0.69	2	1	0.05	1.53 × 10^−12^	0.04	1.28 × 10^−07^	0.01	5.40 × 10^−14^	0.00	0.73
rs34949187	15	89,386,652	*ACAN*	G	A	0.18	2	0.87	0.06	7.65 × 10^−12^	0.03	2.69 × 10^−03^	0.02	1.40 × 10^−37^	0.00	0.42
rs2716212	17	67,503,653	*MAP2K6*	G	A	0.61	2	0.67	0.04	1.18 × 10^−08^	0.06	2.89 × 10^−13^	0.01	3.40 × 10^−14^	0.00	0.27
rs227734	17	54,767,470	*NOG*	T	C	0.3	2	0.98	0.04	7.44 × 10^−09^	0.05	2.66 × 10^−09^	0.02	2.80 × 10^−37^	0.00	0.09
rs823097	1	205,681,370	*NUCKS1*	G	A	0.43	3	0.99	0.04	1.35 × 10^−08^	0.01	0.34	0.01	8.20 × 10^−23^	0.01	5.2 × 10^−03^
rs10933424	2	233,872,408	*NGEF*	T	C	0.89	3	0.91	0.06	2.65 × 10^−09^	−0.01	0.36	0.02	1.20 × 10^−14^	0.00	0.77
rs7633464	3	98,715,823	*DCBLD2*	A	G	0.48	3	1	0.04	5.71 × 10^−12^	0.00	0.85	0.01	4.90 × 10^−10^	0.00	0.29
rs12511230	4	145,471,245	*HHIP*	A	T	0.6	3	1	0.05	5.66 × 10^−18^	0.00	0.96	−0.01	2.70 × 10^−18^	0.00	0.14
rs2545730	5	98,109,985	*RGMB*	G	A	0.52	3	0.98	0.03	3.52 × 10^−08^	0.01	0.26	0.00	0.11	0.00	0.34
rs17138646	5	115,346,245	*AQPEP*	T	G	0.88	3	0.84	0.05	1.28 × 10^−08^	−0.01	0.32	0.00	0.097	0.00	0.99
rs62479589^b^	7	128,406,506	*CALU*	G	A	0.38	3	0.95	0.04	2.42 × 10^−08^	0.01	0.17	0.00	7.4 × 10^−04^	0.00	0.69
rs4744313	9	96,846,061	*PTPDC1*	T	C	0.63	3	0.79	0.04	1.01 × 10^−08^	−0.01	0.25	0.00	0.22	0.01	0.01
rs10962293	9	16,136,648	*C9orf92*	C	T	0.29	3	0.99	0.04	6.58 × 10^−09^	0.00	0.95	0.00	0.82	0.00	0.93
rs1413299	9	101,761,241	*COL15A1*	T	G	0.37	3	0.56	0.04	1.39 × 10^−08^	−0.01	0.13	−0.01	5.30 × 10^−17^	0.01	0.004
rs10739993^c^	9	97,982,669	*FANCC*	C	T	0.59	3	0.98	0.04	1.79 × 10^−08^	0.00	0.94	0.00	7.4 × 10^−04^	0.00	0.15
rs45540840	11	118,486,110	*PHLDB1*	G	A	0.22	3	0.99	0.04	1.87 × 10^−08^	0.00	0.96	0.01	3.60 × 10^−04^	0.00	0.07
rs2260671	12	66,174,909	*HMGA2*	A	G	0.08	3	1	0.1	8.19 × 10^−19^	0.00	0.98	0.01	3.30 × 10^−04^	0.00	0.58
rs1809360	15	68,189,737	*SKOR1*	C	T	0.57	3	1	0.05	1.12 × 10^−13^	0.00	0.65	0.00	0.84	−0.02	2.90 × 10^−14^
rs117564279	15	81,224,038	*CEMIP*	A	G	0.02	3	0.85	0.15	1.35 × 10^−08^	0.06	0.07	0.00	0.67	−0.01	0.14
rs7179372	15	67,036,441	*SMAD6*	G	A	0.2	3	1	0.05	4.12 × 10^−10^	0.01	0.44	0.01	2.70 × 10^−11^	−0.01	0.02
rs62070652	17	29,221,277	*ATAD5*	C	T	0.27	3	0.98	0.05	2.62 × 10^−14^	0.02	0.05	0.04	1.60 × 10^−166^	0.00	0.4
rs8097746	18	46,640,782	*DYM*	T	C	0.59	3	1	0.06	9.12 × 10^−20^	0.01	0.14	0.02	4.80 × 10^−51^	0.00	0.02
rs34717890	19	46,400,443	*MYPOP*^d^	T	C	0.12	3	1	0.1	1.24 × 10^−27^	−0.01	0.25	0.00	0.18	0.00	0.39
rs61648765	19	46,381,864	*FOXA3*^d^	C	G	0.78	3	1	0.07	2.11 × 10^−19^	0.00	0.9	0.01	4.40 × 10^−04^	0.00	0.17
rs34687269	9	119,484,132	*ASTN2*^d^	A	T	0.52	Pal	Pal	0.07	3.96 × 10^−32^	0.07	2.32 × 10^−19^	0.01	6.20 × 10^−23^	0.00	0.71

Each conditionally independent mJSW SNP is assigned to a cluster according to HOA effect by MR-Clust. The probability for it being a member of that cluster is given. Each SNP effect and *P*-value is given for a GWAS of mJSW, HOA, standing height and BMI.

C.Gene – closest gene, mJSW – minimum joint space width, Pal – Palindromic SNP, SNP – single nucleotide polymorphism, HOA – hip osteoarthritis, *P* – *P*-value.

a Proxy SNP rs687621 (r2 = 0.98).

b rs6954748 (r2 = 0.97).

c rs7854570 (r2 = 0.99).

d rs7869550, rs76248879, rs34687269 mapped to *PAPPA-ASTN2* locus, and rs61648765 and rs34717890 mapped to *FOXA3-MYPOP* locus based on a 1 mb sliding window approach.

### Genetic correlation

LDSC provided estimates of genetic correlation. A strong genetic correlation was seen between mJSW_DXA_ (N = 38,175) versus mJSW_X-ray_ (N = 12,570) (r_g_ 0.87 [95% CI 0.59, 1.14]). While the SNP heritability z-score for mJSW_DXA_ was 10.8, the SNP heritability z-score for mJSW_X-ray_ was 3.3, which is below the threshold of 4 that is suggested for reliable LDSC estimates.^22^ There was a moderate correlation between mJSW_combined_ (mJSW_DXA_ and mJSW_X-ray_ combined) versus height (r_g_ 0.28 [0.22, 0.33]) and between mJSW_DXA_ versus height (r_g_ 0.34 [0.28, 0.39]). There was weak genetic correlation between mJSW and BMI and HOA, confidence intervals excluding zero in the case of mJSW_DXA_ and BMI (r_g_ 0.08 [0.03, 0.14]) and HOA (r_g_ 0.14 [0.04, 0.25]), but including zero for mJSW_combined_ versus BMI (r_g_ 0.06 [0.01, 0.12]) and versus HOA (r_g_ 0.10 [−0.01, 0.21]) (Supplementary Table S3).

### Mendelian randomisation and MR-cluster

**Fig. 2:**
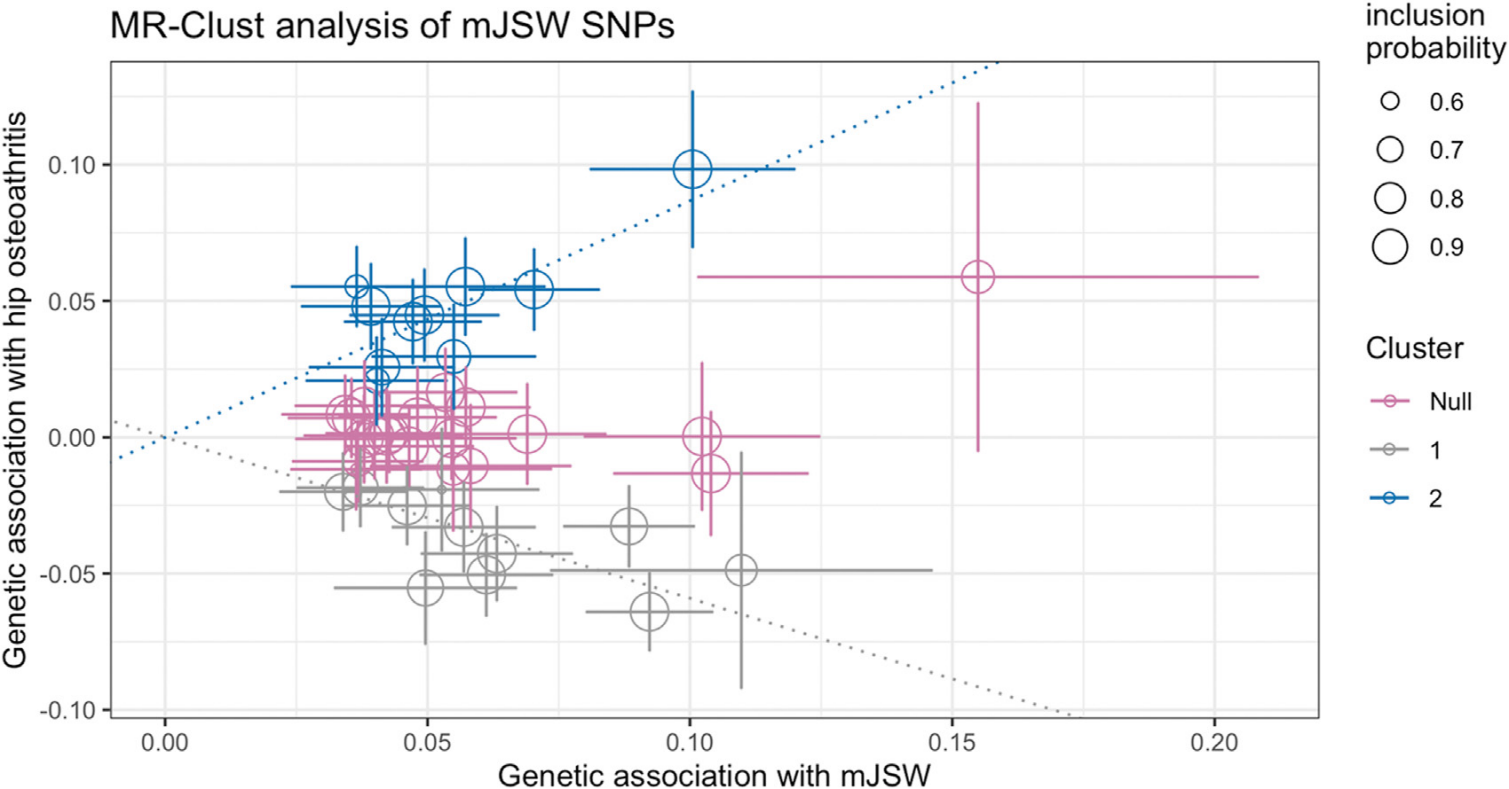
MR-Clust results. Each independent minimum joint space width (mJSW) single nucleotide polymorphism (SNP) is plotted comparing their mJSW and hip osteoarthritis (HOA) effects. Three clusters are identified: Cluster one SNPs show a protective effect on HOA with increasing mJSW, Cluster two SNPs show an increasing risk of HOA with increasing mJSW and the null cluster SNPs show no effect on HOA.

To examine the causal relationship between mJSW and osteoarthritis, we performed a two sample MR. 41 of the 42 independent mJSW lead SNPs were used as genetic instruments (mean F-statistic = 59, range 30–222, Supplementary Table S4). Rs34687269 was not included in the MR analyses because its alleles are palindromic. Despite good instrument strength, two sample MR showed no causal effect of mJSW on HOA (IVW: Odds Ratio (OR) 0.98 [95% CI 0.82–1.18], MR Egger: OR 0.69 [0.40–1.18] and Weighted Median: OR 0.98 [0.88–1.09]) (Supplementary Fig. S4). Subsequent cluster analysis of the mJSW genetic instruments displayed three distinct clusters, with two sample MR used to quantify each cluster's effects: (i) Cluster one SNPs (n = 11) were associated with a higher mJSW and a decreased risk of HOA (IVW: OR 0.55 [95% CI 0.49–0.62]); (ii) Cluster two SNPs (n = 10) were associated with both greater mJSW and an increased risk of HOA (IVW: OR 2.40 [2.04–2.82]); and (iii) Cluster three SNPs (n = 20) had no clear association with HOA (IVW: OR 1.03 [0.95–1.11]) (Table 2, Fig. 2, and Supplementary Figs. S5–S7). Heterogeneity of SNP effects between mJSW and HOA identified by cluster analysis illustrated why no net causal effect between these traits was detected. To further understand these SNP clusters, SNP associations with other traits were investigated.

**Table 2:** Two sample Mendelian randomisation results.

Exposure	Outcome	SNPs	IVW		MR egger		Weighted median		Simple mode		Weighted mode	
			OR (95% CI)	*P*	OR (95% CI)	*P*	OR (95% CI)	*P*	OR (95% CI)	*P*	OR (95% CI)	*P*
mJSW	HOA	41	0.98 (0.82–1.18)	0.87	0.69 (0.40–1.18)	0.18	0.98 (0.88–1.09)	0.72	0.97 (0.79–1.20)	0.80	0.93 (0.76–1.13)	0.49
Cluster 1	HOA	11	0.55 (0.49–0.62)	2.47 × 10^−25^	0.59 (0.42–0.85)	0.02	0.56 (0.49–0.64)	1.61 × 10^−16^	0.58 (0.45–0.73)	1.94 × 10^−03^	0.52 (0.40–0.67)	3.64 × 10^−04^
Cluster 2	HOA	10	2.40 (2.04–2.82)	2.98 × 10^−26^	1.97 (1.07–3.63)	0.06	2.36 (1.98–2.81)	8.62 × 10^−21^	2.40 (1.82–3.17)	1.99 × 10^−04^	2.33 (1.90–2.84)	3.71 × 10^−05^
Cluster 3	HOA	20	1.03 (0.95–1.11)	0.47	1.01 (0.79–1.29)	0.91	1.01 (0.91–1.12)	0.86	1.00 (0.81–1.23)	0.98	0.99 (0.83–1.18)	0.90

Exposure	Outcome	SNPs	Beta (95% CI)	*P*	Beta (95% CI)	*P*	Beta (95% CI)	*P*	Beta (95% CI)	*P*	Beta (95% CI)	*P*
Cluster 1	Height	11	0.06 (−0.02 to 0.14)	0.16	0.20 (−0.05 to 0.45)	0.16	0.01 (−0.01 to 0.03)	0.17	0.01 (−0.01 to 0.03)	0.45	0.01 (0.00–0.03)	0.16
Cluster 2	Height	10	0.25 (0.14–0.36)	1.36 × 10^−05^	−0.07 (−0.40 to 0.26)	0.69	0.10 (0.07–0.13)	2.75 × 10^−11^	0.30 (0.11–0.50)	0.01	0.11 (0.08–0.13)	1.01 × 10^−05^
Cluster 3	Height	20	0.09 (−0.01 to 0.19)	0.07	0.09 (−0.22 to 0.40)	0.58	0.02 (−0.01 to 0.04)	0.22	0.02 (−0.01 to 0.06)	0.25	0.01 (−0.01 to 0.03)	0.22

IVW – inverse variance weighted, MR – Mendelian randomisation, OR – odds ratio, mJSW – minimum joint space width, SNP – single nucleotide polymorphism, CI – confidence interval, *P* – *P*-value, HOA – hip osteoarthritis.

### Trait look-ups and SNP prioritisation

The 42 independent mJSW-associated SNPs were examined in previous GWAS of HOA, height, and BMI (Table 1). SNPs in Cluster one (n = 11), which were associated with a decreased risk of HOA with increasing mJSW, showed mixed associations with height; MR showed limited evidence of a small causal effect on height overall (IVW: β 0.06 [95% CI −0.02, 0.14]) (Table 2). SNPs in Cluster two (n = 10), which were associated with a higher HOA risk with increasing mJSW, all (except one for which a proxy SNP was not found) showed strong and consistent positive associations with height and some evidence of association with BMI, and MR showed a strong causal effect of these SNPs on height (IVW: β 0.25 [95% CI 0.14, 0.36]) (Table 2 and Supplementary Figs. S8 and S9). Colocalisation analysis revealed that two SNPs in Cluster two (near *BMP6* and *MAP2K6*) shared common signals with height (Bayesian colocalisation: PP 99% and 97%, respectively) (Supplementary Table S5).

The palindromic SNP (rs34687269) showed strong positive associations with mJSW, HOA and height, in keeping with rs76248879, a Cluster two SNP that was also situated close to the *ASTN2* locus (Table 1). There is a clear null cluster outlier (rs117564279) in Fig. 2, which shows a strong mJSW effect (Linear regression: β 0.15, *P* 1.35 × 10^−8^) and weaker HOA (Linear regression: β 0.06, *P* 0.07) effect. Interestingly, it has no association with height (Linear regression: β 0.002, *P* 0.67). Rs117564279 is a rare allele with a MAF 0.02 (Table 1).

### Identification of candidate osteoarthritis pathogenesis genes

SNPs in Cluster one, which were thought to increase HOA risk through reduced mJSW and hence are potential targets for chondro-protective therapies, were assessed further. Colocalisation was used to compare GWAS signals between mJSW and HOA. Loci closest to *TGFA, COL27A1, C8orf34* and *SLBP* showed strong evidence of a shared signal (Bayesian colocalisation: PP 100%, 100%, 99% & 97%, respectively). No other loci showed such evidence (Table 3).

**Table 3:** Cluster one candidate gene identification.

C.Gene	RSID	HOA Beta	HOA *P*	HOA Coloc (PP)	MAGMA–top genes (*P*-value)
*TGFA*	rs7571789	−0.06	5.32 × 10^−18^	1.00	*TGFA* (4.83 × 10^−17^)
*SUPT3H*	rs10948155	−0.05	9.17 × 10^−11^	0.03	SUPT3H (6.93 × 10^−15^), RUNX2 (1.55 × 10^−12^)
*EPC1*	rs76164690	−0.06	1.59 × 10^−07^	0.00	EPC1 (3.41 × 10^−06^)
*C8orf34*	rs7846438	−0.04	1.48 × 10^−06^	0.99	C8orf34 (3.83 × 10^−09^)
*AP3D1*	rs34656141	−0.03	2.34 × 10^−05^	0.40	DOT1L (3.11 × 10^−15^)
*COL27A1*	rs4979342	−0.03	9.40 × 10^−05^	1.00	COL27A1 (2.21 × 10^−12^)
*SLBP*	rs2236996	−0.03	6.74 × 10^−04^	0.97	TMEM129 (3.45 × 10^−12^), TACC3 (9.78 × 10^−11^), SLBP (1.05 × 10^−14^)
*MN1*	rs2106973	−0.02	6.95 × 10^−03^	0.16	N/A
*ALDH1A2*	rs11857461	−0.02	0.01	0.25	ALDH1A2 (8.45 × 10^−09^)
*TIAM2*	rs35199713	−0.05	0.03	0.00	N/A
*EGFR*	rs17172430	−0.02	0.10	0.05	N/A

Cluster one SNPs were labelled with the closest gene. They were looked up in a HOA GWAS and their beta and *P*-value is given in the columns “HOA Beta” and “HOA” *P* respectively. Colocalisation was used to assess whether mJSW and HOA GWAS signals share common genetic causal variant in a given region with the posterior probability (PP) reported (H4: both traits are associated and share a single causal variant). Gene set analysis (MAGMA) was used to identify further candidate genes. The *P*-value threshold was 2.65 × 10^−06^ (linear regression). eQTL signals were assessed in GTEx using LocusFocus to conduct colocalisation with posterior probabilities reported. Cultured fibroblasts or if not present the tissue with the largest evidence of expression are reported.

C.Gene – closest gene, HOA – hip osteoarthritis, PP – posterior probability, Coloc – colocalisation, GTEx – genotyping expression project, N/A – not applicable, MAGMA – generalised gene-set analysis of GWAS data.

Subsequently, attempts were made to identify the underlying causal gene responsible for the SNP association. MAGMA assigned *TGFA, SUPT3H-RUNX2, C8orf34, EPC1, COL27A1, SLBP*-*TMEM129-TACC3, ALDH1A2* and *DOT1L* as candidate genes (Table 3). *TGFA* and *SUPT3H* mJSW association signals colocalised with GTEx expression in amygdala and basal ganglia respectively, but not in fibroblasts (Supplementary Table S6). The outlier SNP (rs117564279) with the largest effect size near *CEMIP* was also examined in GTEx and colocalised with eQTL SNPs in skeletal muscle (PP 0.98). RegulomeDB suggested the SNPs nearest to *TGFA, AP3D1, EGFR* and *TIAM2* were non-coding regulatory regions with probability scores > 0.5 (Supplementary Table S7). Colocalisation between mJSW SNPs and human cartilage eQTL data provided no further gene-SNP evidence for our prioritised SNPs (Supplementary Table S8). However, it did show evidence of colocalisation for two null cluster SNPs; rs62479589 with *OPN1SW* in both highly and less degraded cartilage (Bayesian colocalisation: PP 97% & 90% respectively) and rs823097 with *RAB7L1* in highly degraded cartilage (Bayesian colocalisation: PP 96%) (Supplementary Table S8).

### Gene ontology biological process annotations

PANTHER and FUMA GeNE2FUNC analyses showed that three Cluster two SNPs (which mapped to *ACAN*, *NOG*, *BMP6*) overlapped with skeletal system morphogenesis and development (Supplementary Tables S9 and S10). However, MAGMA gene-set analysis returned no results suggesting little evidence for overlap with any of the gene sets tested.

## Discussion

In the largest GWAS of hip mJSW to date, we identified 42 conditionally independent SNPs, mapping to 39 loci. Overall MR analysis revealed little evidence for a causal effect of mJSW on HOA risk. However, cluster analysis identified three groups of SNPs with distinct effects. One cluster comprised 11 SNPs which increase mJSW leading to a decrease in HOA risk. In contrast, a second cluster comprised 10 SNPs which increased mJSW but led to an increase in HOA risk. The latter set of SNPs was also related to height, a known risk factor for HOA. A null cluster comprised 20 SNPs with no association with HOA risk. Taken together, these findings suggest that SNPs associated with mJSW may exert distinct effects on HOA risk according to whether this is instrumented by SNPs which are also related to height.

Of the 11 loci in Cluster one, which were protective for HOA with increasing mJSW, *TGFA, C8orf34, COL27A1* and *SLBP-TMEM129-TACC3* colocalised with the same causal signal for HOA in a large-scale HOA GWAS. The present findings suggest that these previously identified loci cause HOA through reduced cartilage thickness, suggesting potential utility as therapeutic targets for chondro-protective therapy. *TGFA* was implicated in mJSW by a previous much smaller GWAS and is known to be involved in endochondral bone formation.^17^^,^^34^, ^35^, ^36^ Likewise, *COL27A1* is established in cartilage regulation and formation, and mutations are associated with osteochondrodysplasias in humans such as Steel syndrome which feature early hip dislocations and OA.^37^^,^^38^ There is little known about *C8orf34* regulation of joint tissues such as cartilage but it has been implicated in vertebral disc disease.^39^ In addition, MAGMA suggested *TMEM129, SLBP* and *TACC3* might be the genes responsible for the association with mJSW at rs2236996 locus but this was not supported by eQTL findings. *TMEM129* mutations can lead to facial dysmorphias such as Wolf-Hirschhorn syndrome and has been suggested to be a genetic risk factor for OA through disrupted protein degradation in the endoplasmic reticulum.^40^, ^41^, ^42^

The other loci identified in Cluster one, *SUPT3H-RUNX2, AP3D1, EPC1, MN1, ALDH1A2, TIAM2* and *EGFR* did not colocalise with corresponding HOA GWAS signals but nonetheless showed at least a nominal HOA association. The *SUPT3H-RUNX2* locus was identified in the previous mJSW GWAS and has been implicated in chondrocyte and osteoblast differentiation respectively.^17^^,^^36^ MAGMA suggested *EPC1* as a candidate for rs76164690, however this signal did not colocalise with eQTL expression in fibroblasts. Pigment epithelium derived factor (PEDF) is the product of the *EPC1* gene and is known to be anti-angiogenic. Previously PEDF has been shown to be preferentially expressed in OA cartilage contributing to OA pathogenesis by upregulating matrix degrading factors.^43^^,^^44^ Whilst there was evidence of an association between rs34656141 with eQTL expression for *AP3D1, DOT1L* and *AMH* in fibroblasts these signals did not colocalise. *DOT1L* was previously implicated in mJSW in a smaller GWAS and is known to regulate cartilage homeostasis and protect against OA.^17^^,^^45^ Anti-Mullerian Hormone, the product of *AMH*, is associated with knee OA in women.^46^ *ALDH1A2, TIAM2* and *EGFR* showed less evidence of an association with HOA, that said, *ALDH1A2* and *EGFR* have previously been identified as potential treatment targets for OA.^47^^,^^48^ Less is known about *AP3D1, TIAM2* and *MN1* in the context of cartilage and HOA.

One locus showed a SNP effect of increased mJSW and HOA risk that was not associated with height; rs117564279 (*CEMIP*) is a rare variant with a MAF 0.02 and large effect size for both mJSW and HOA (β 0.15 & β 0.06 respectively). *CEMIP* is the closest gene and showed colocalisation between eQTL expression (skeletal muscle) and the mJSW GWAS signal. *CEMIP* has recently been shown to be expressed in cartilage from osteoarthritic joints, and to induce a fibrosis type response within chondrocytes.^49^ Therefore, *CEMIP* warrants further investigation to understand if altered expression leads to thicker more fibrous cartilage which in turn could lead to a wider joint space and a higher risk of HOA.

**Fig. 3:**
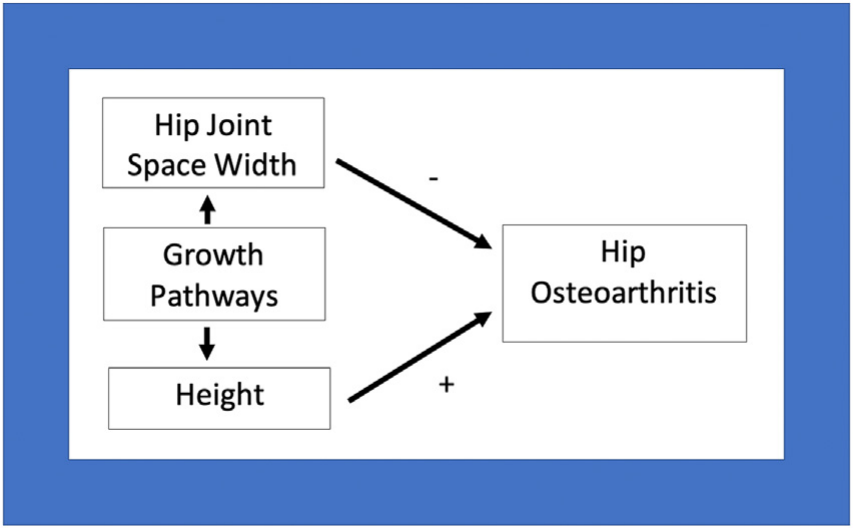
A directed acyclic graph to represent the proposed relationships between hip minimum joint space width, height and hip osteoarthritis.

The opposing effects of SNPs in clusters one and two, as shown by the MR analyses of each cluster, presumably lead to a net null effect of mJSW on HOA. This may help to explain why mJSW, when examined observationally, displays little or no associations with HOA and symptoms, yet a decreased mJSW is often seen clinically in severely symptomatic individuals.^7^ Our observation that Cluster two SNPs are related to both height and HOA is consistent with previous findings that height GWAS signals overlap with OA.^50^^,^^51^ This also corresponds with findings from observational studies that taller individuals are at an increased risk of HOA.^8^^,^^52^ Whereas Cluster two SNPs are related to height, mJSW GWAS results showed little attenuation following height adjustment. Therefore, Cluster two SNPs appear to increase HOA risk through co-association with greater height, although height itself does not appear to be on the causal pathway for mJSW, suggesting the role of an intermediary growth-related mechanism (Fig. 3). Consistent with this suggestion, gene ontology annotation suggested that three Cluster two SNPs (*ACAN*, *NOG*, *BMP6*) have a role in skeletal development. Extra-skeletal endocrine actions that influence growth might also play a role, given two loci, *PAPPA* and *PIK3R1,* are involved in the action of IGF-1 and insulin.^53^^,^^54^

The strengths of this study include its large sample size which has afforded the power to identify 35 loci not previously known to be associated with mJSW. In addition, by combining a GWAS meta-analysis with other genetic analyses such as LDSC, MR and MR-Clust we have been able to tease out different causal pathways related to mJSW. Arguably, the main limitation was our combination of DXA and X-ray based measures for deriving mJSW. Though DXA-derived mJSW represents a different method, the finding of an inverse relationship with rHOA (see Supplementary Methods) provides face validity. However certain differences exist in mJSW measurements using these methods. For example, unlike DXA scans where only the superior joint space can be evaluated, mJSW can also be measured on X-rays at other sites. That said, X-ray based mJSW measurements were based solely on the superior joint space in SOF and MrOS. In contrast, in RS, mJSW measurements were also obtained laterally, axially and medially, with the smallest value used. Despite these differences, genetic correlation between mJSW obtained using these two methods was relatively high, albeit the mJSW_X-ray_ GWAS was underpowered for LDSC analysis.

In terms of other limitations, as this is a GWAS of individuals with European ancestry this limits generalisability to other ancestries. In addition, there were some methodological differences in how GWAS was performed in the different cohorts, reflecting the fact that these had been initially undertaken as part of separate studies. Finally, there was limited evidence of colocalisation between GWAS and eQTL data which hinders the identification of effector genes. However, it is increasingly recognised that many true GWAS signals fail to colocalise with eQTL signals.^55^^,^^56^

In conclusion, we present findings from a GWAS meta-analysis of hip mJSW which identified 39 loci. Subsequently, we showed that mJSW SNPs act on HOA in two distinct clusters; those that decrease HOA risk with increasing mJSW and those that increase HOA risk via increasing mJSW. We postulate the first group of SNPs may act via cartilage mediated pathways, suggesting possible utility as targets for chondroprotective therapies. In contrast, the latter group of SNPs are associated with greater height and likely act through growth-related mechanisms which require further clarification.

## Contributors

BGF and MF have verified the underlying data and take responsibility for the findings in the manuscript.

Study design: MF, BGF; Data Analysis: MF, BGF, RE, KAF, ML, AH, FRS.; Interpretation of results: all authors; Replication data: CGB, DSE.; Manuscript drafting: MF, BGF, JPK, JT.; Manuscript reviewing and editing: all authors. In addition, all authors have read and approved the final version of the manuscript.

## Data sharing statement

The summary minimum joint space width summary statistics have been uploaded to the GWAS Catalog (https://www.ebi.ac.uk/gwas/). The UK Biobank mJSW data from this study will be available in a forthcoming data release. Users must be registered with UK Biobank to access their resources (https://bbams.ndph.ox.ac.uk/ams/).

## Declaration of interests

BGF is a member of the Versus Arthritis Research Advisory Group. DE reports grants income from NIH/NIA U24AG051129. FRS reports Wellcome Trust payments to fund meetings and travel. ML reports a CSIRO PhD Top Up scholarship. CL have a patent Image processing apparatus and method for fitting a deformable shape model to an image using random forest regression voting. This is licensed with royalties to Optasia Medical. NH reports consultancy fees and honoraria from UCB, Amgen, Kyowa Kirin, Thornton Ross, Consilient. AEH started working for Novo Nordisk A/S after drafting this manuscript. GDS reports funding from the MRC for the MRC Integrative Epidemiology Unit at the University of Bristol (MC_UU_00011/1). JHT reports funding from the MRC, a payment from Liverpool Medical Institution and he is a member of the Royal Osteoporosis Society grants panel.
