# Supplementary material for: The identification of distinct protective and susceptibility mechanisms for hip osteoarthritis: findings from a genome-wide association study meta-analysis of minimum joint space width and Mendelian randomisation cluster analyses
Source: eBioMedicine. 2023 Aug 22;95:104759. doi: 10.1016/j.ebiom.2023.104759 (PMC10470292; doi:10.1016/j.ebiom.2023.104759)
Supplement: Supplementary Figs. S1–S9 [file mmc2.docx]

## Supplementary Figure 1.1-1.42 Locus zoom plots for 42 leading mJSW SNPs


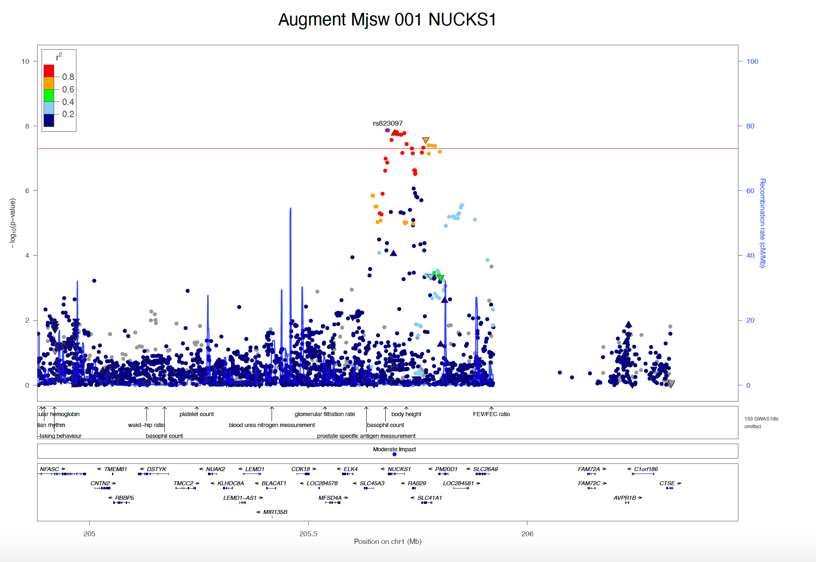


## Supplementary figure 1.1


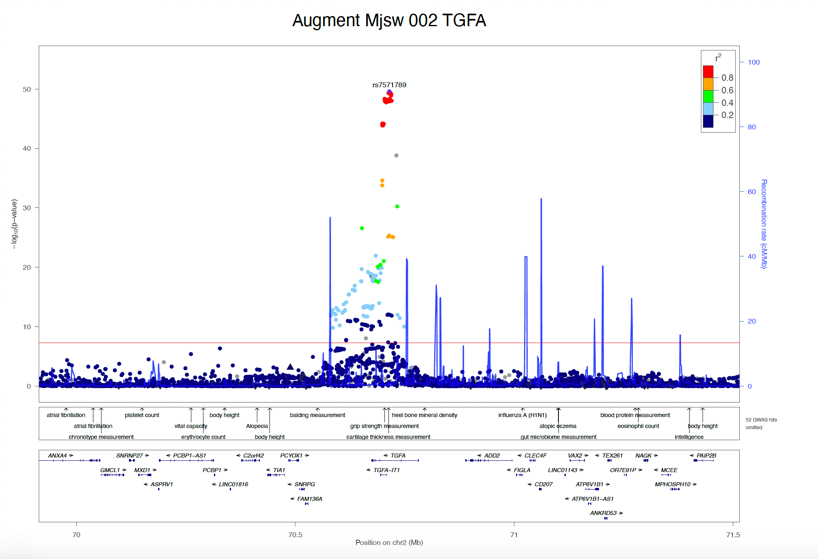


## Supplementary figure 1.2


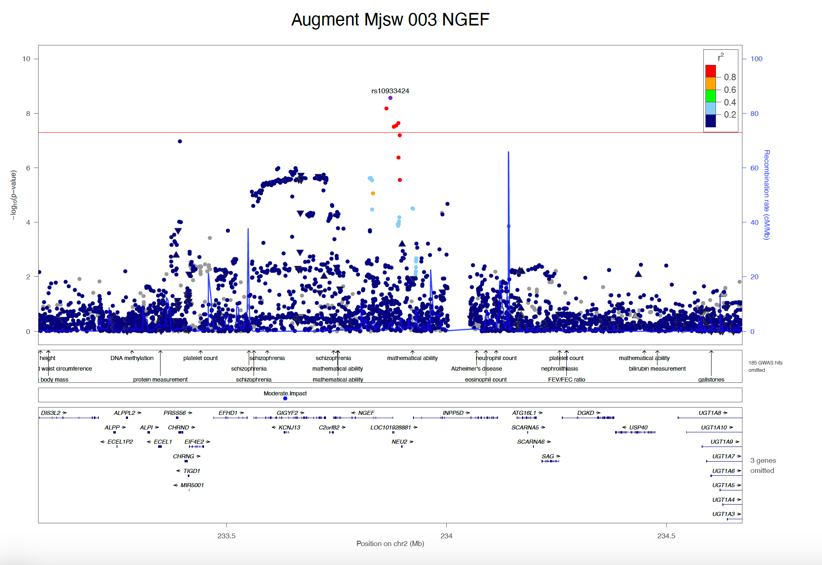


## Supplementary figure 1.3


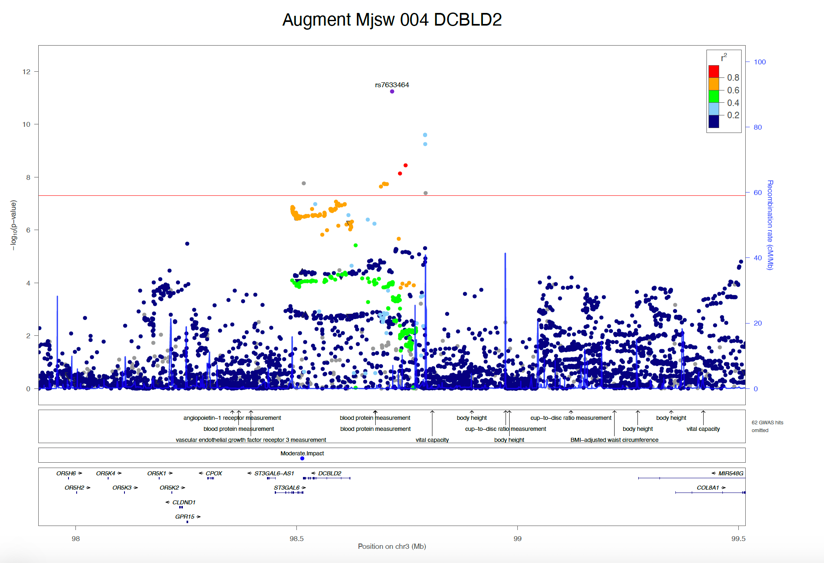


## Supplementary figure 1.4


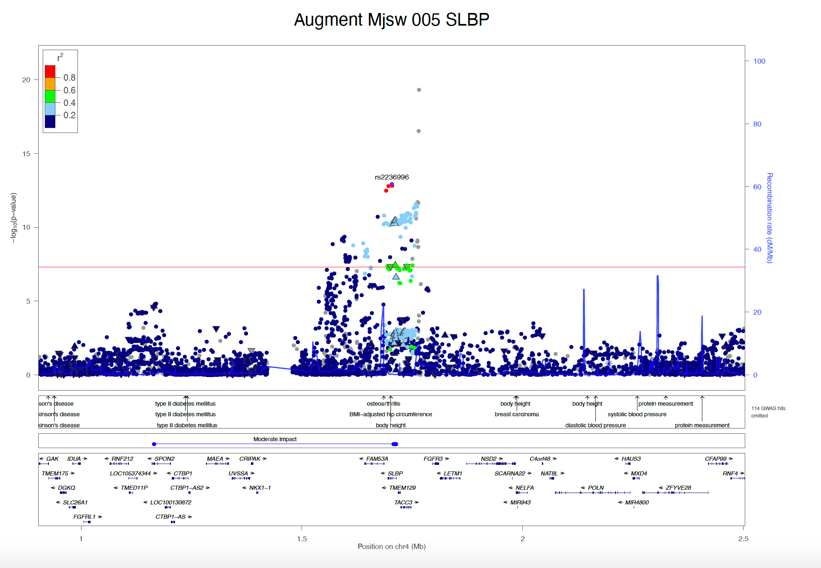


## Supplementary figure 1.5


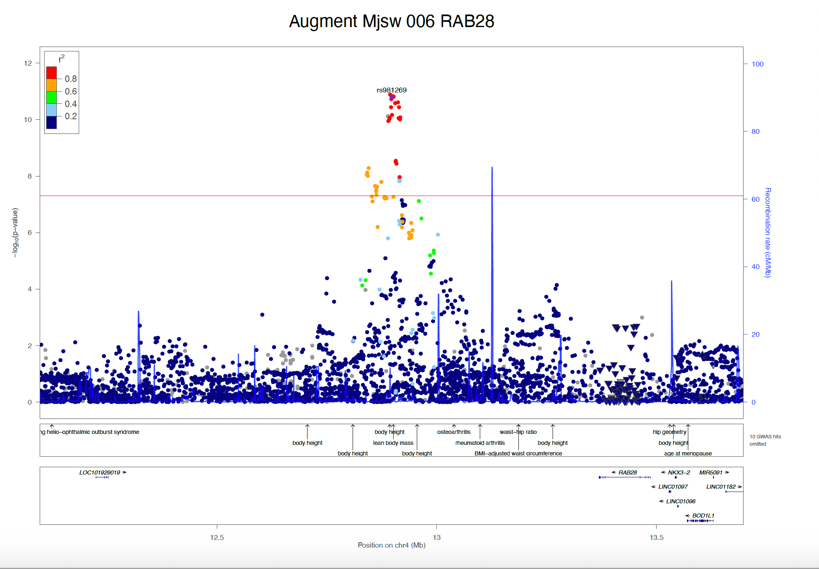


## Supplementary figure 1.6


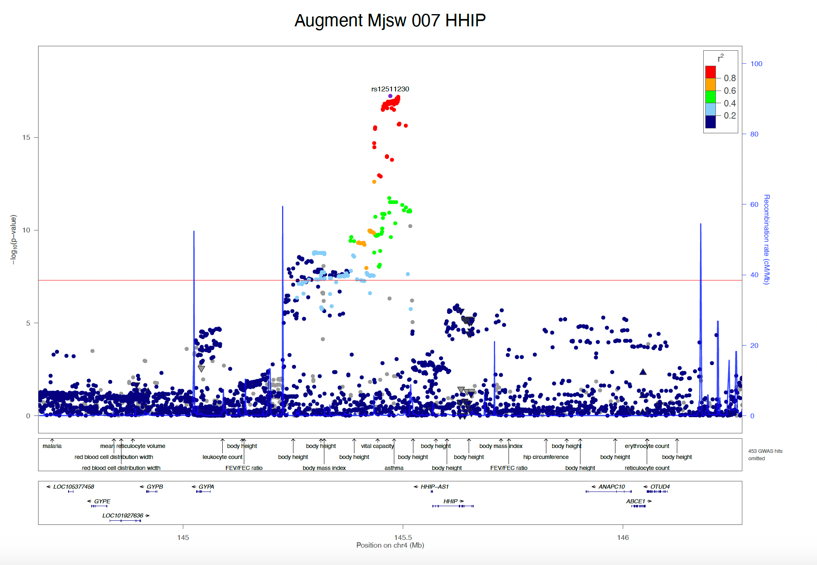


## Supplementary figure 1.7


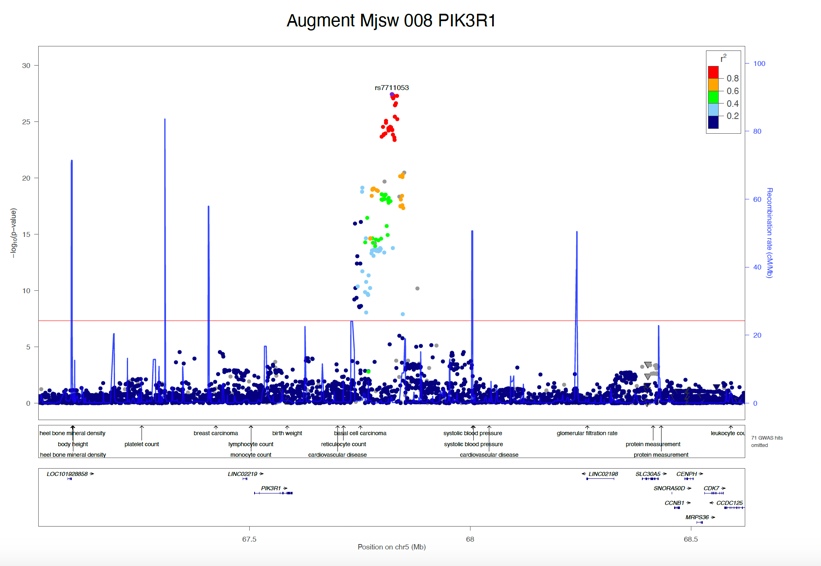


## Supplementary figure 1.8


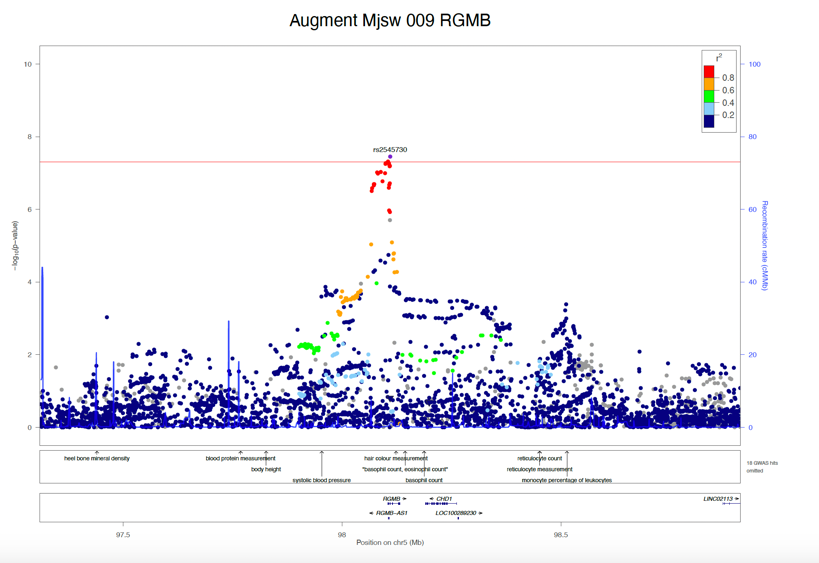


## Supplementary figure 1.9


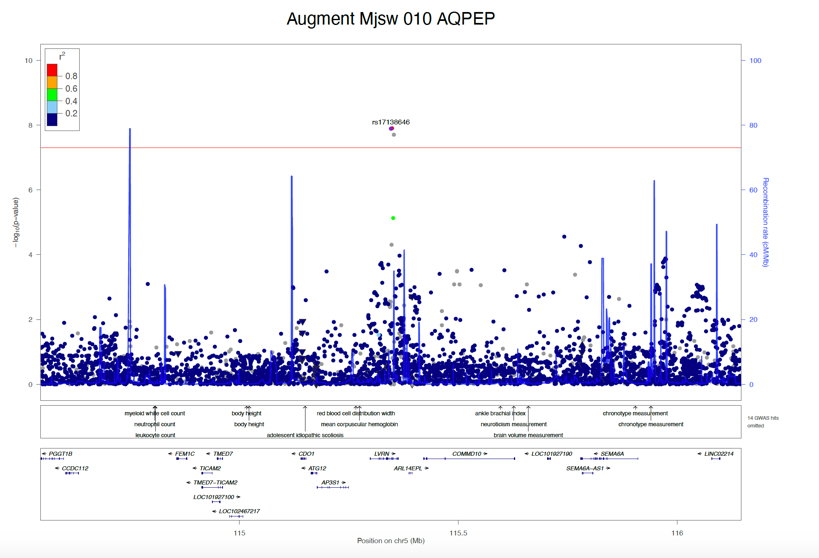


## Supplementary figure 1.10


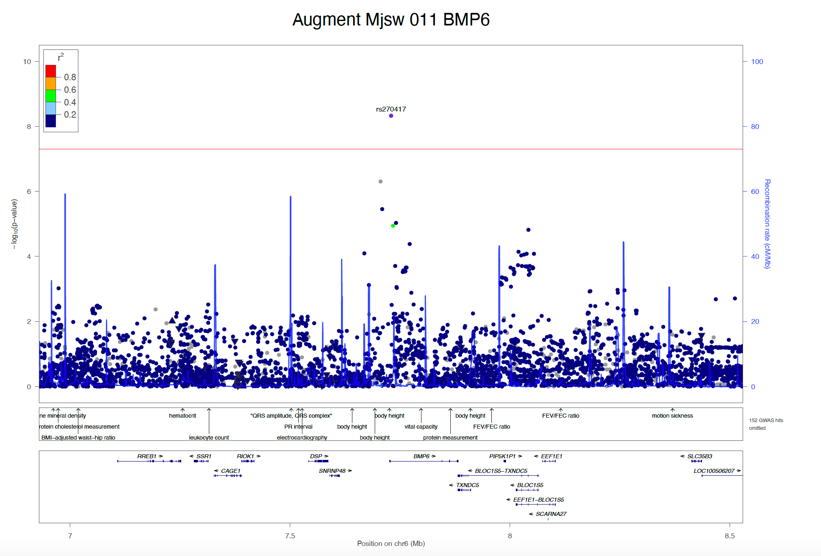


## Supplementary figure 1.11


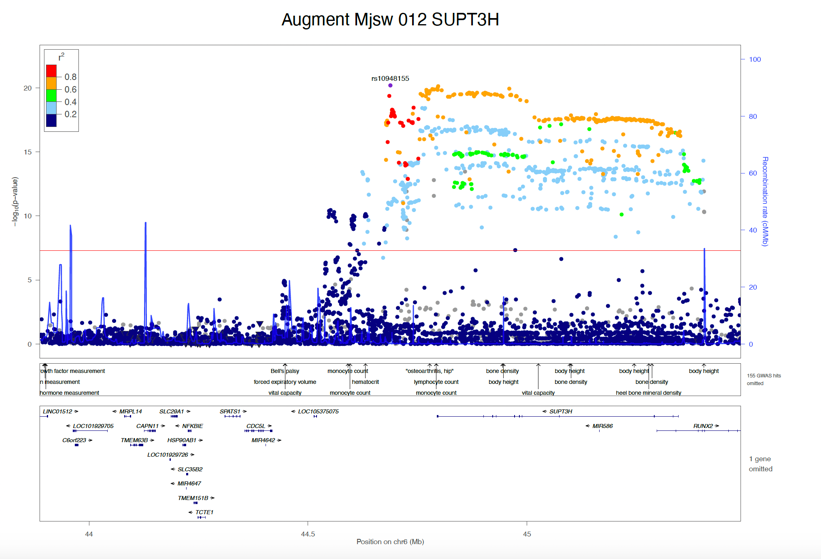


## Supplementary figure 1.12


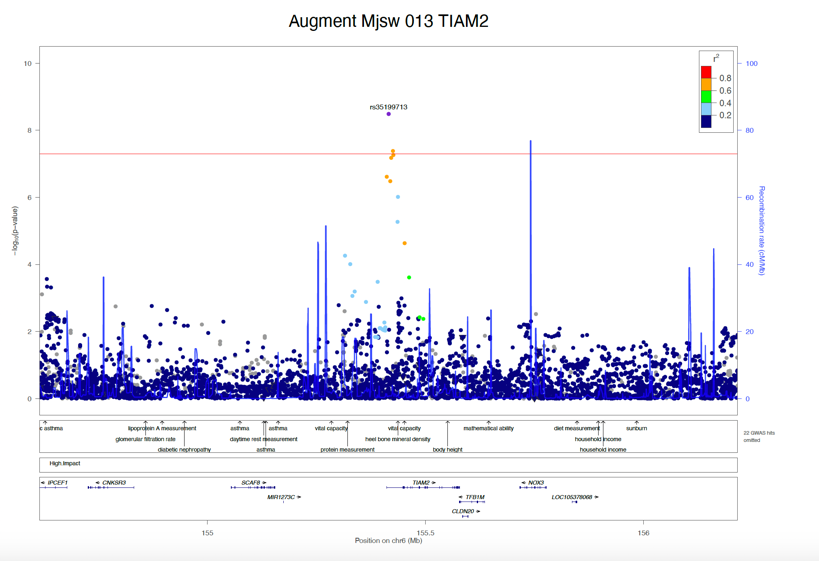


## Supplementary figure 1.13


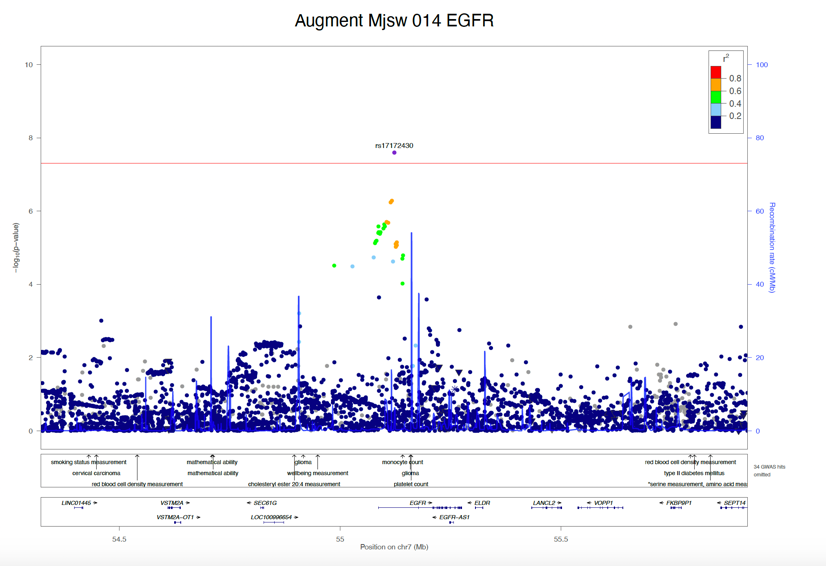


## Supplementary figure 1.14


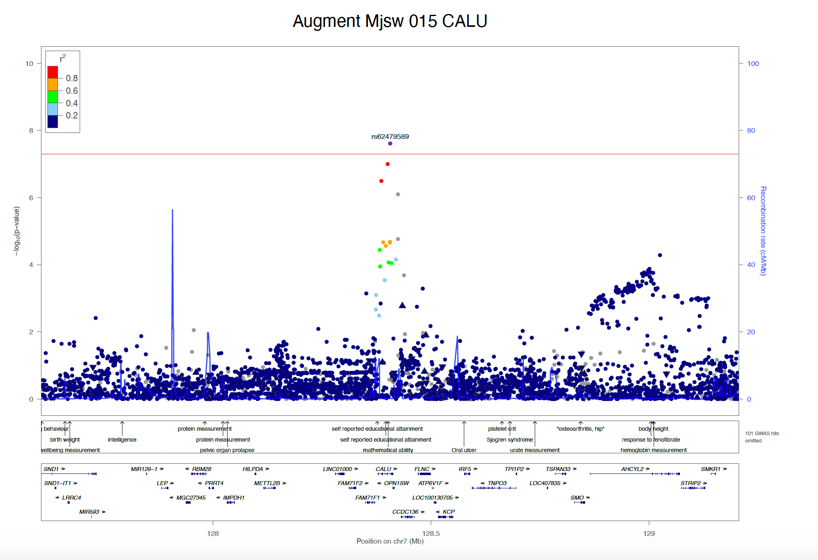


## Supplementary figure 1.15


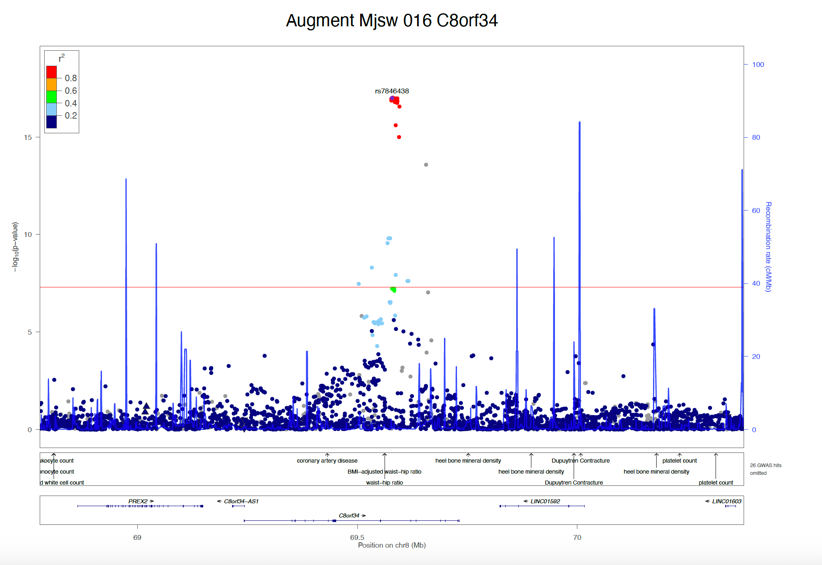


## Supplementary figure 1.16


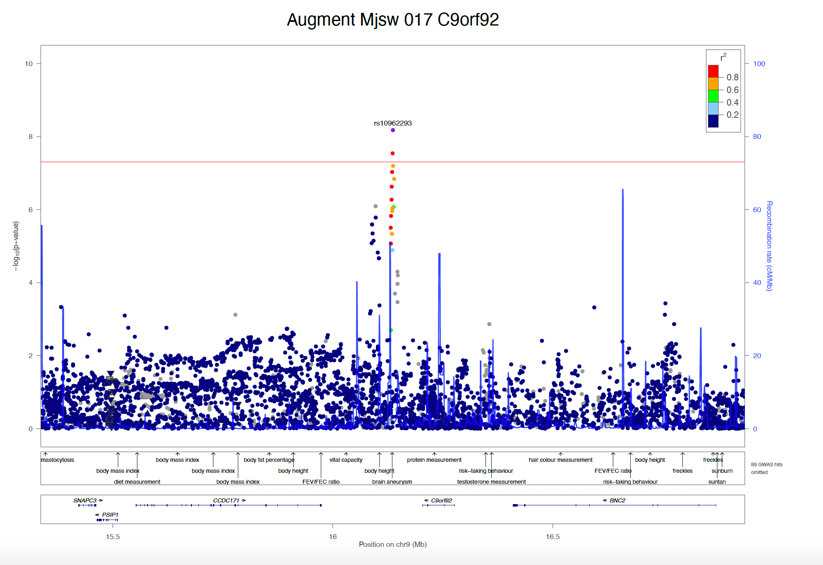


## Supplementary figure 1.17


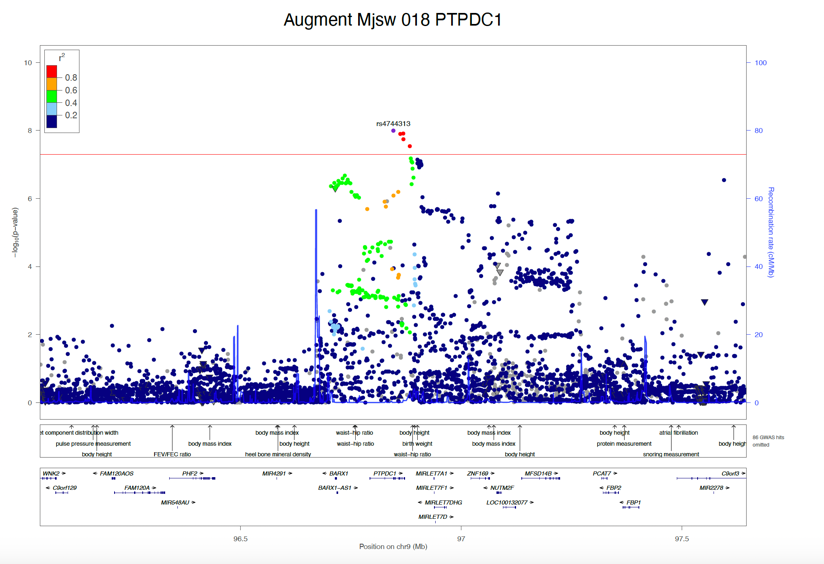


## Supplementary figure 1.18


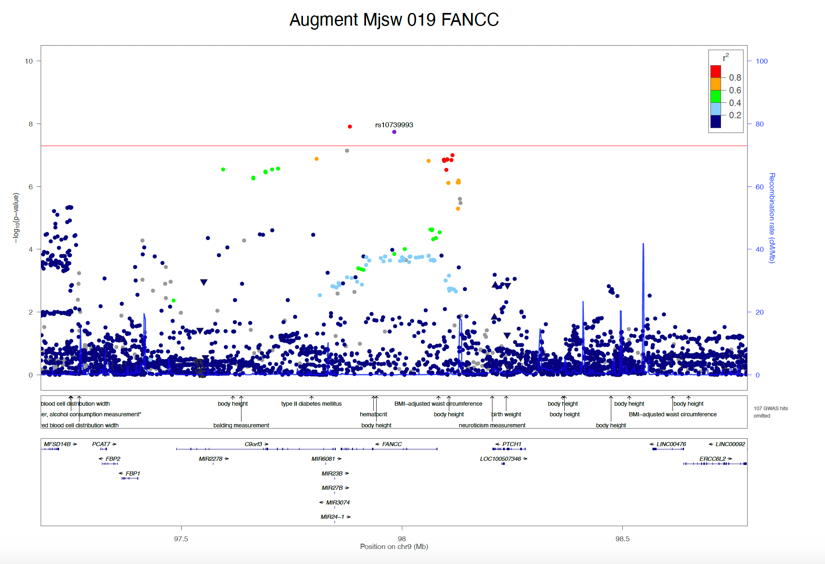


## Supplementary figure 1.19


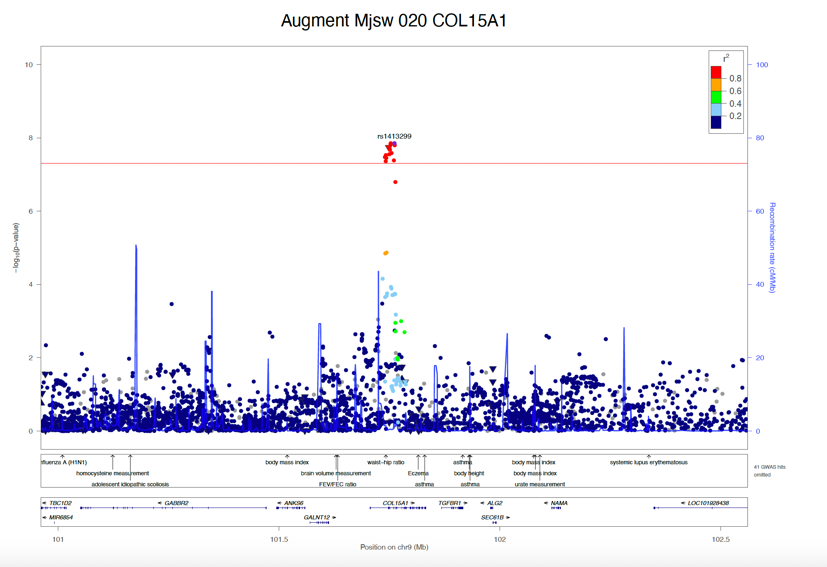


## Supplementary figure 1.20


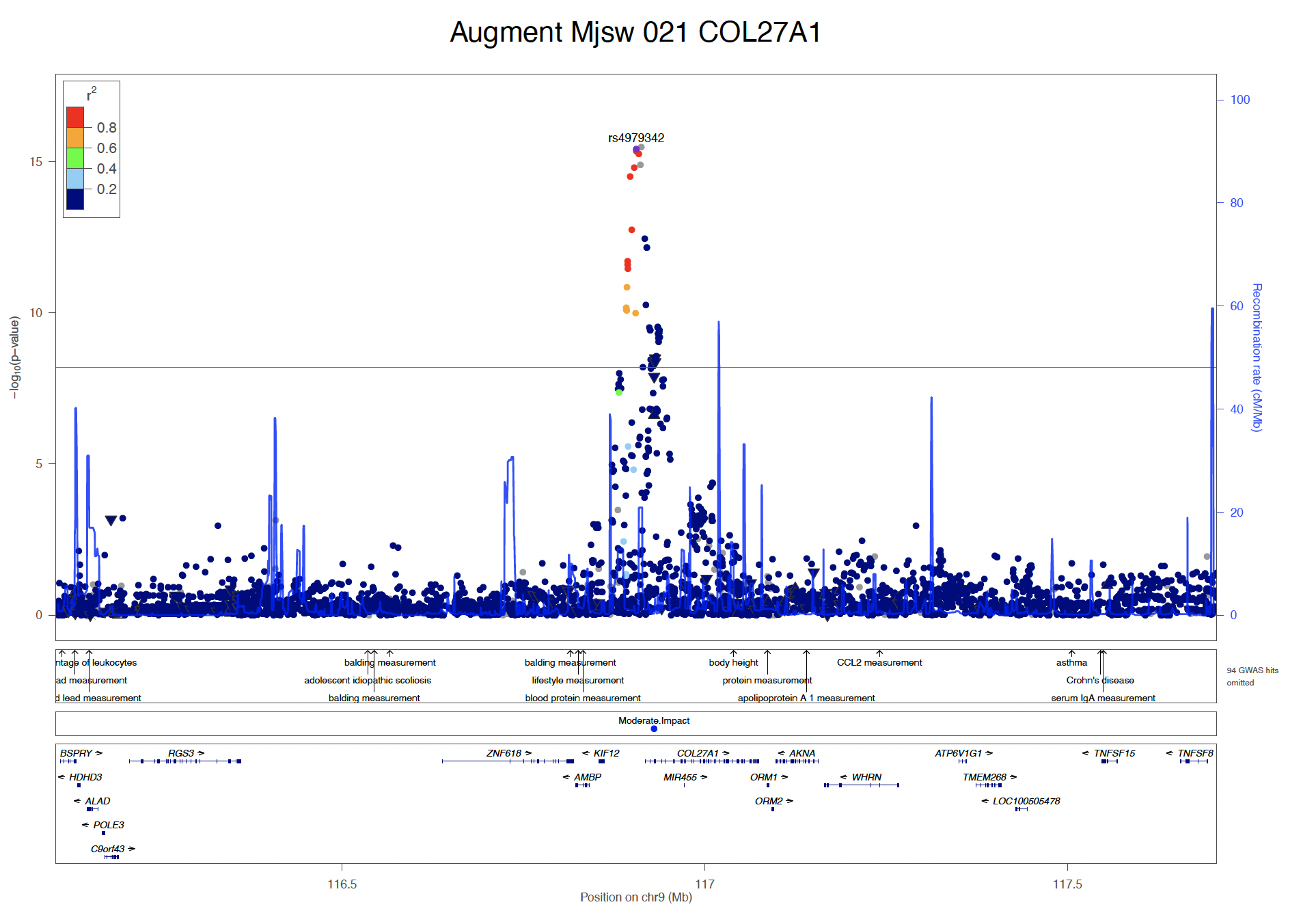


## Supplementary figure 1.21


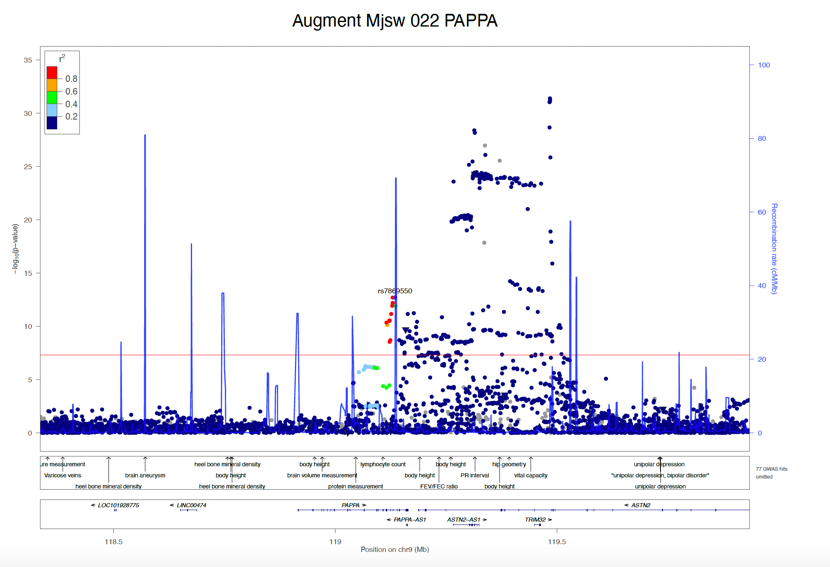


## Supplementary figure 1.22


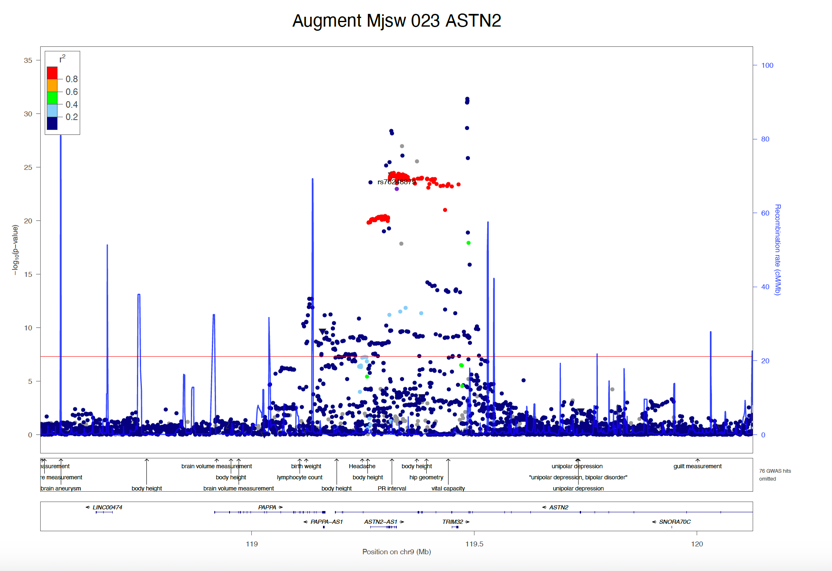


## Supplementary figure 1.23


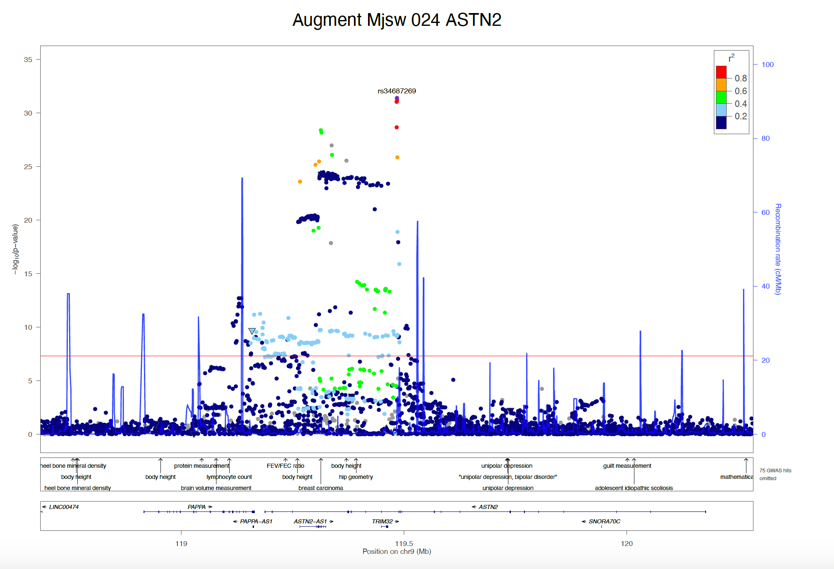


## Supplementary figure 1.24


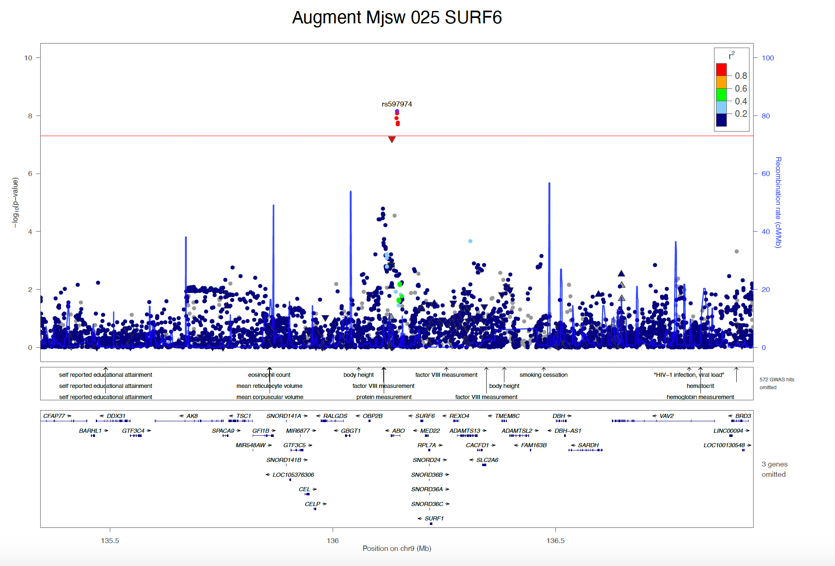


## Supplementary figure 1.25


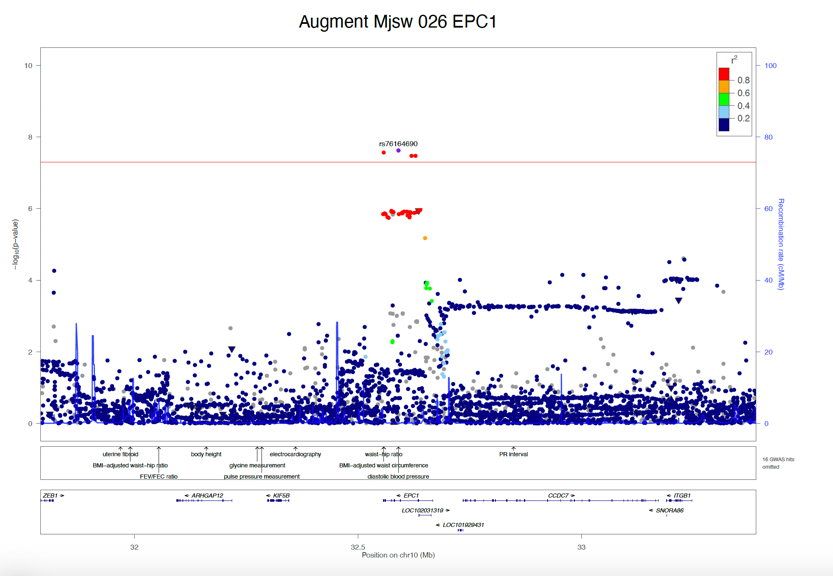


## Supplementary figure 1.26


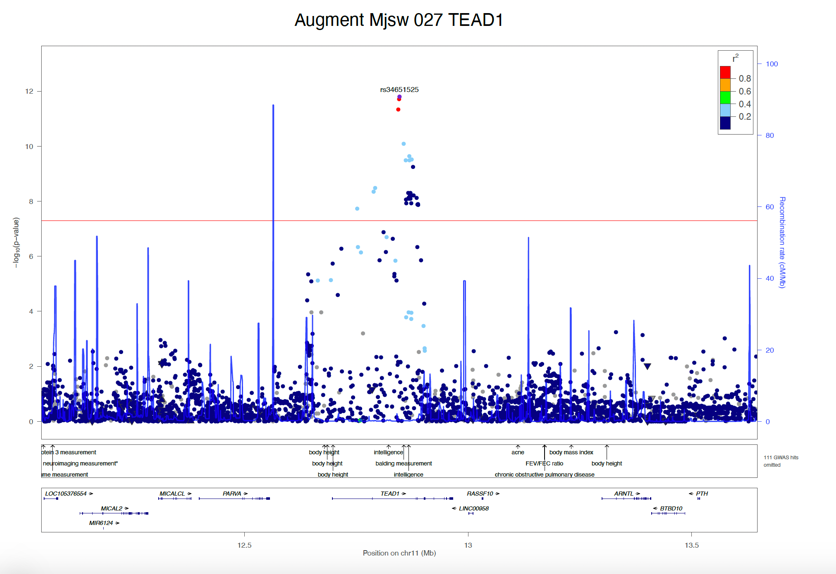


## Supplementary figure 1.27


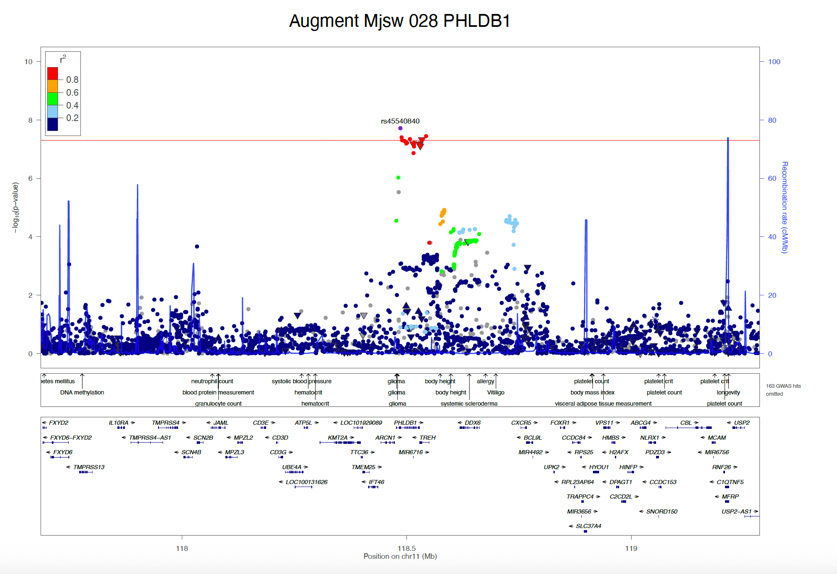


## Supplementary figure 1.28


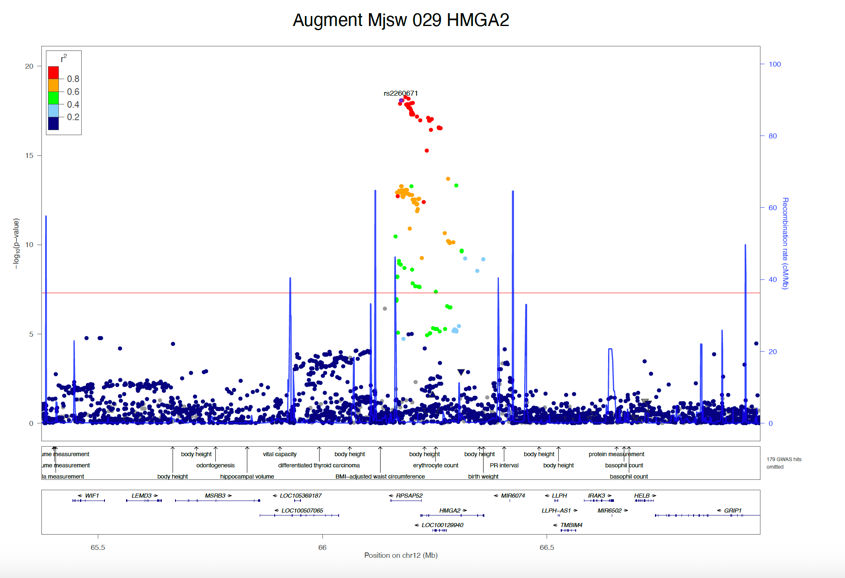


## Supplementary figure 1.29


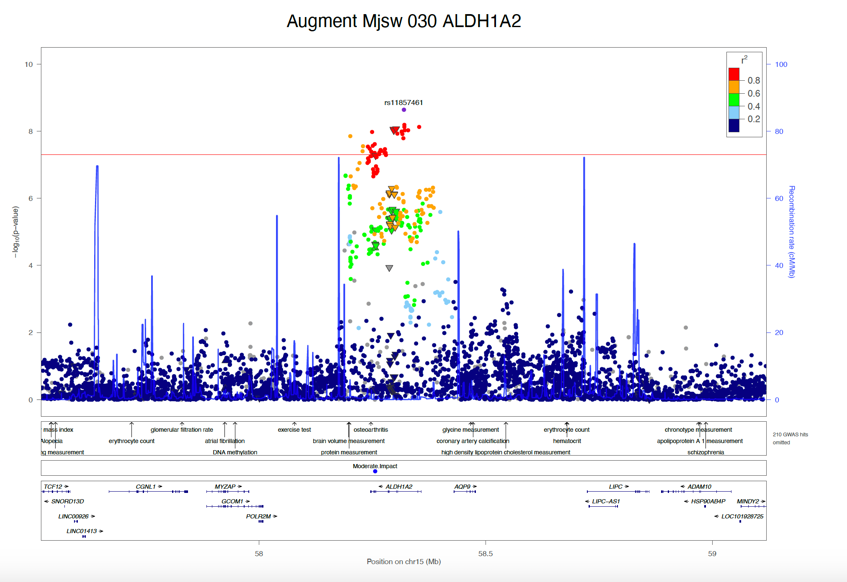


## Supplementary figure 1.30


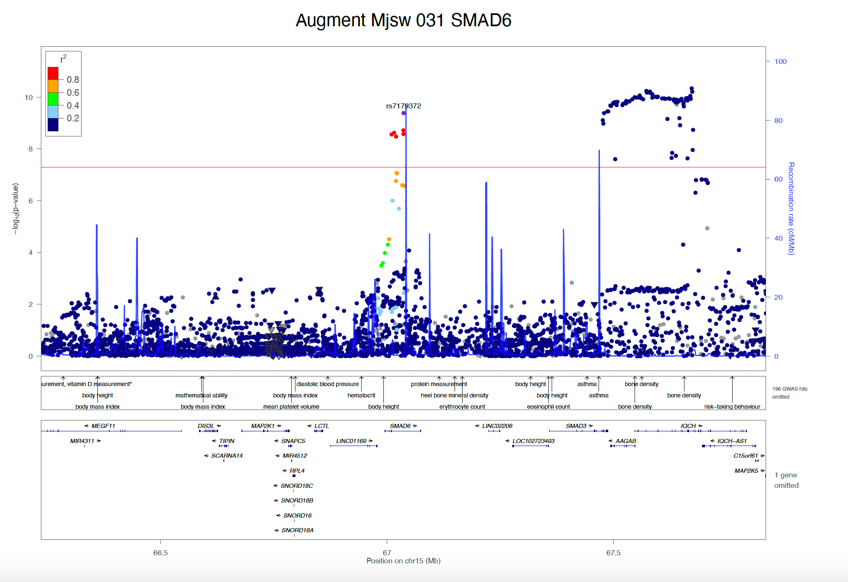


## Supplementary figure 1.31


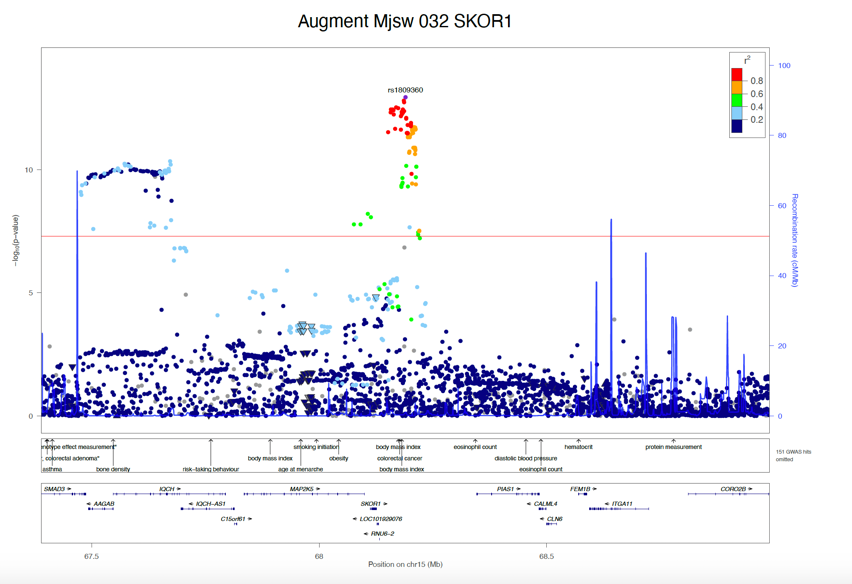


## Supplementary figure 1.32


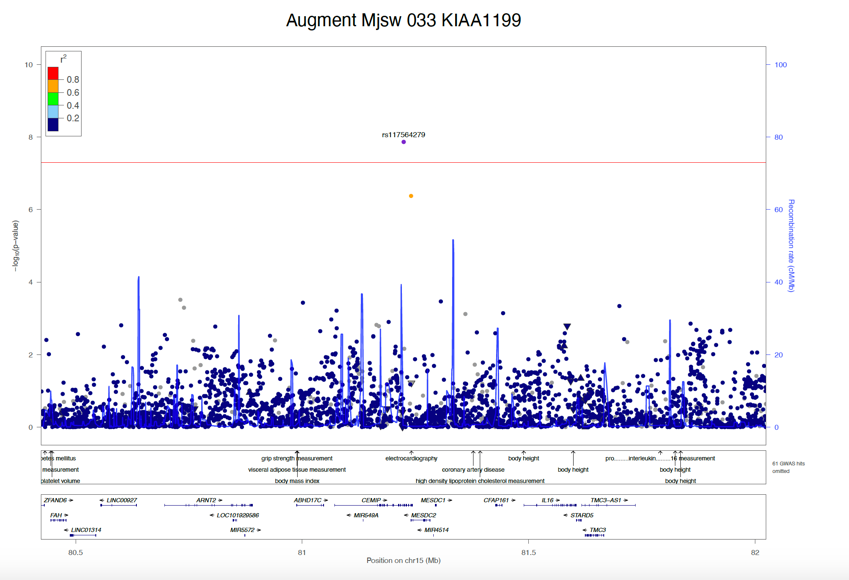


## Supplementary figure 1.33


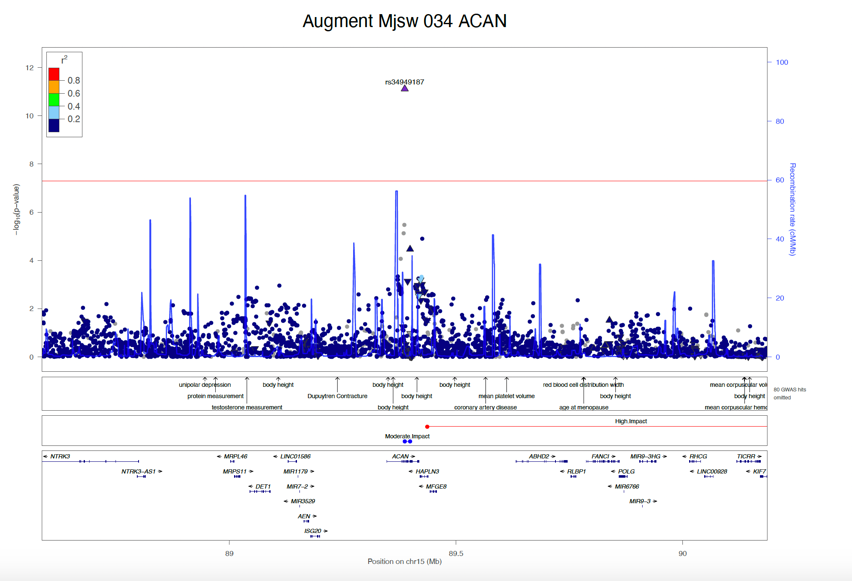


## Supplementary figure 1.34


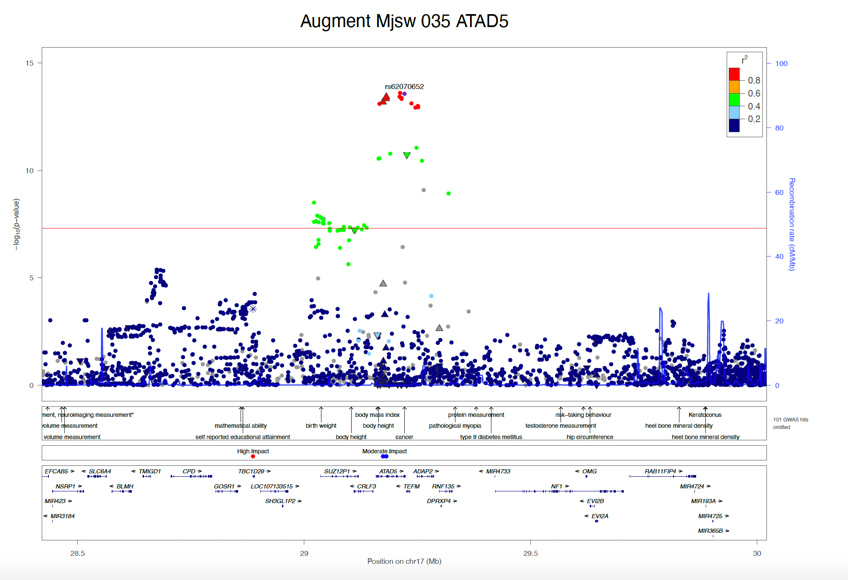


## Supplementary figure 1.35


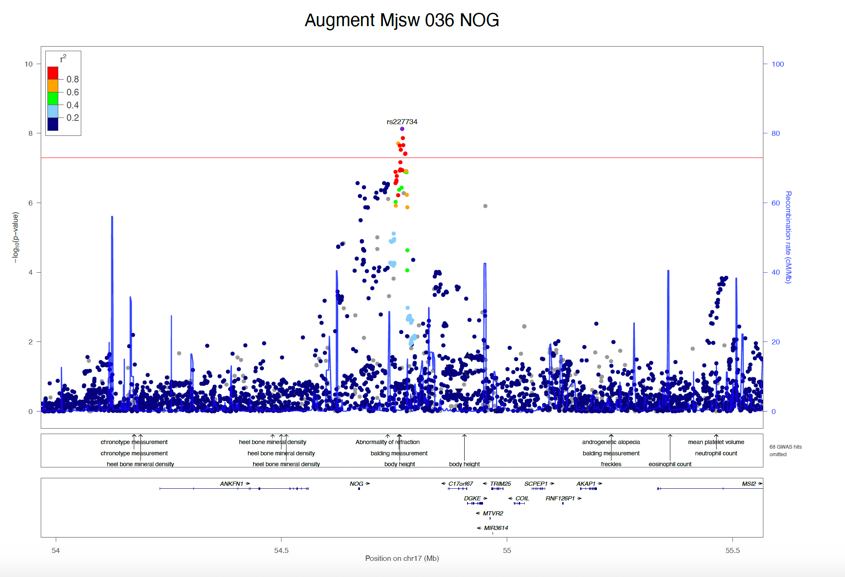


## Supplementary figure 1.36


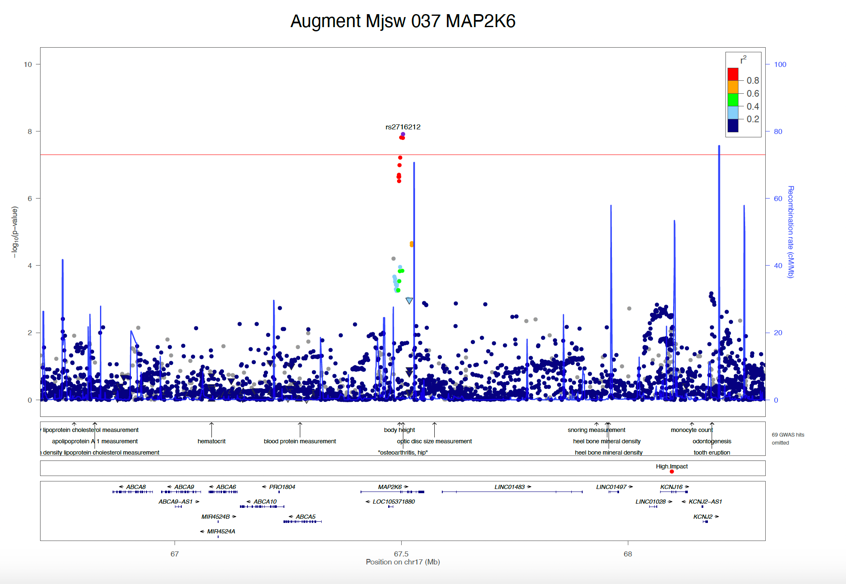


## Supplementary figure 1.37


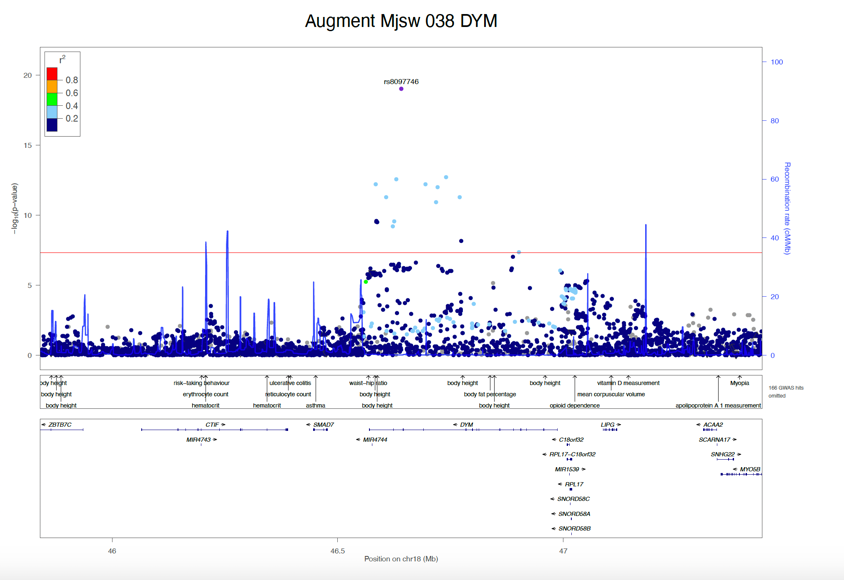


## Supplementary figure 1.38


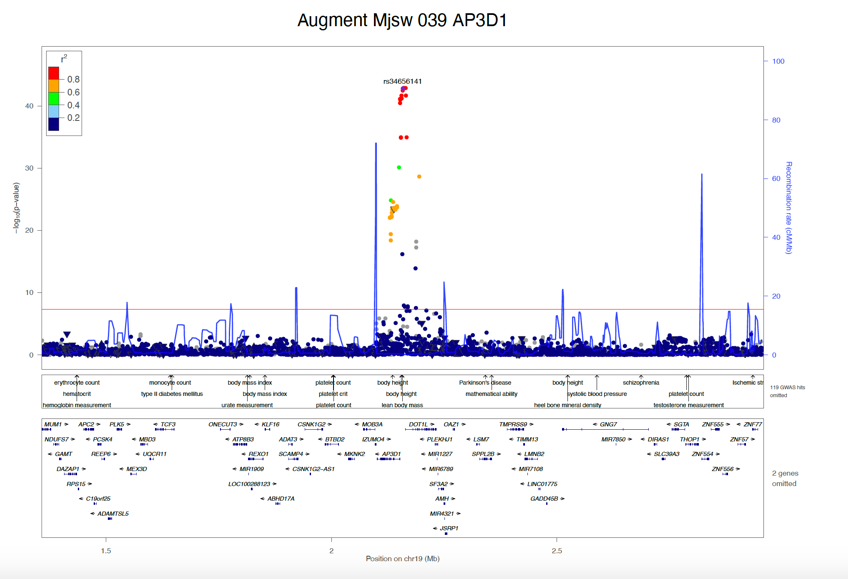


## Supplementary figure 1.39


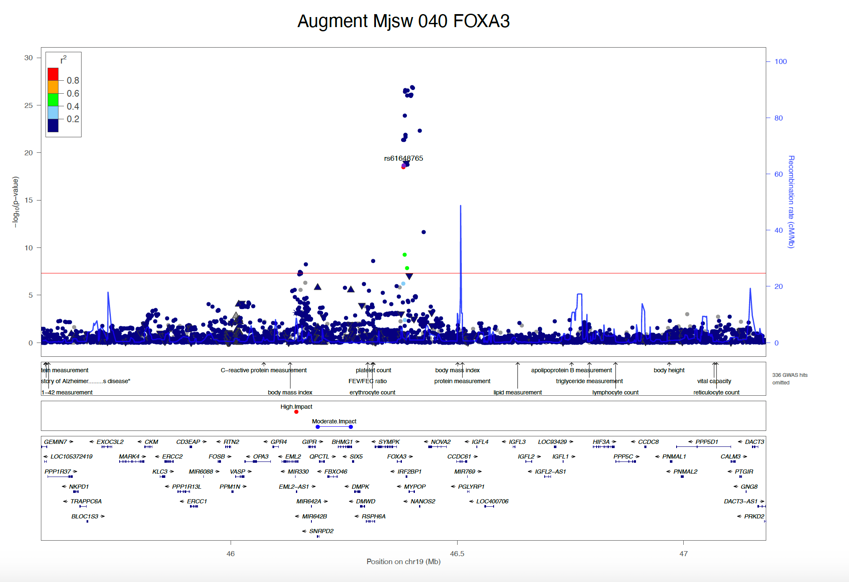


## Supplementary figure 1.40


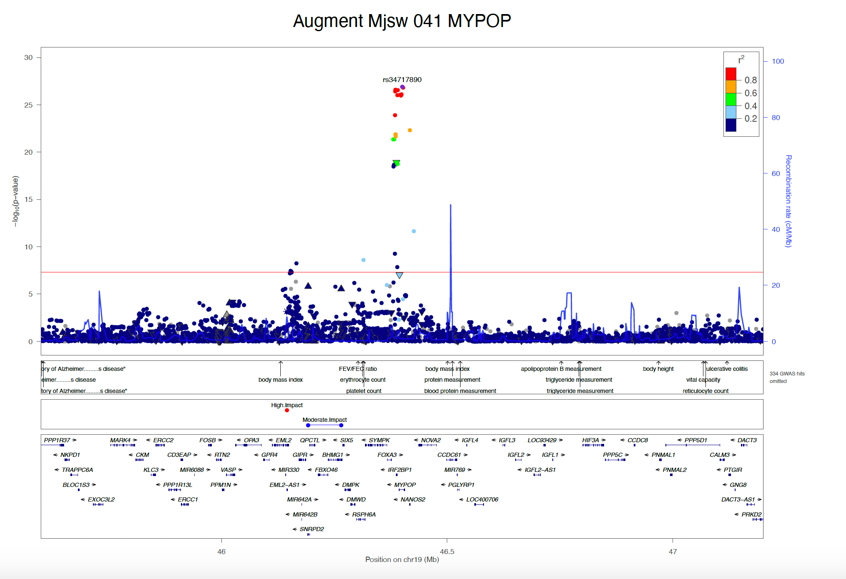


## Supplementary figure 1.41


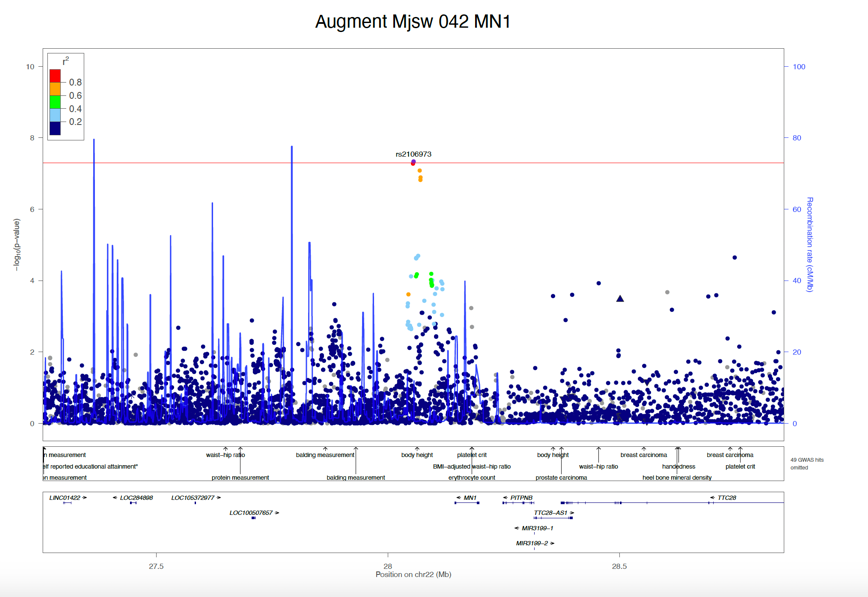


## Supplementary figure 1.42


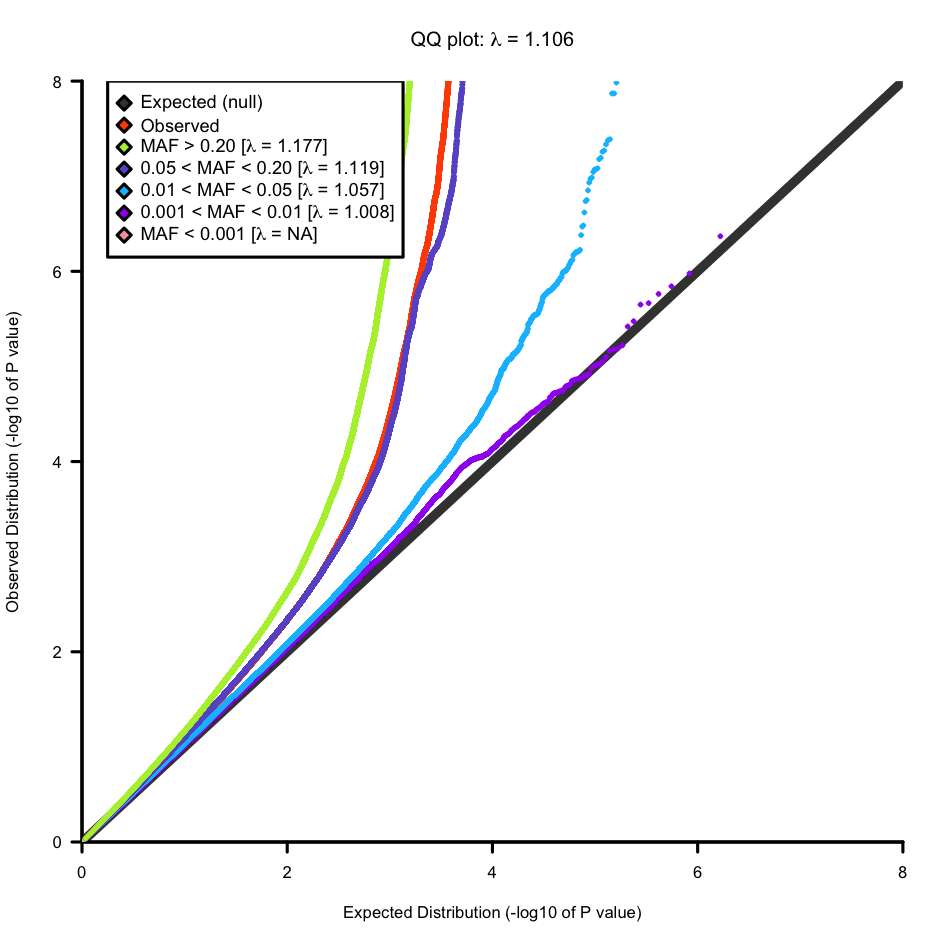


## Supplementary Figure 2: QQ plot of minimum joint space meta-analysis


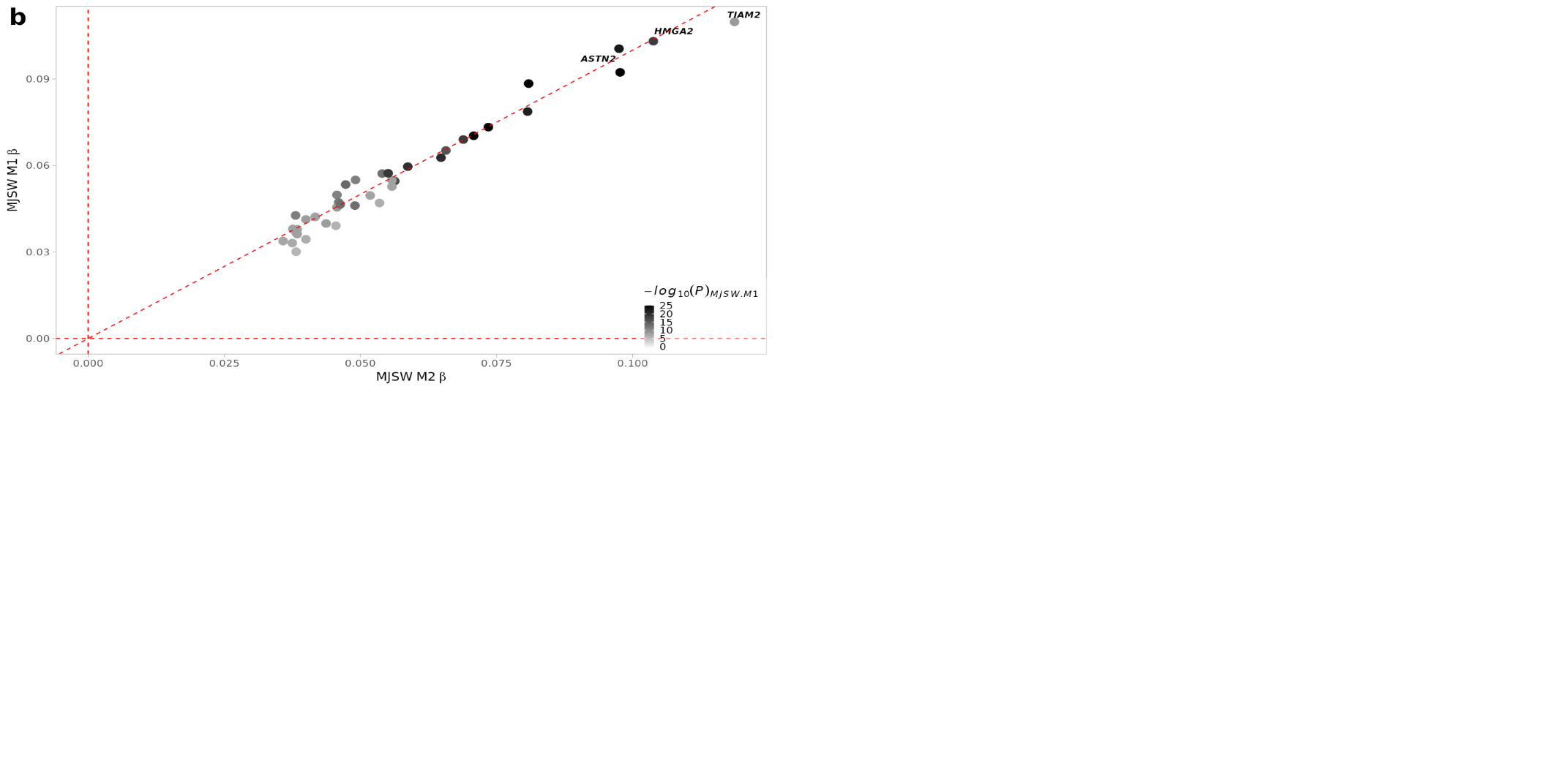

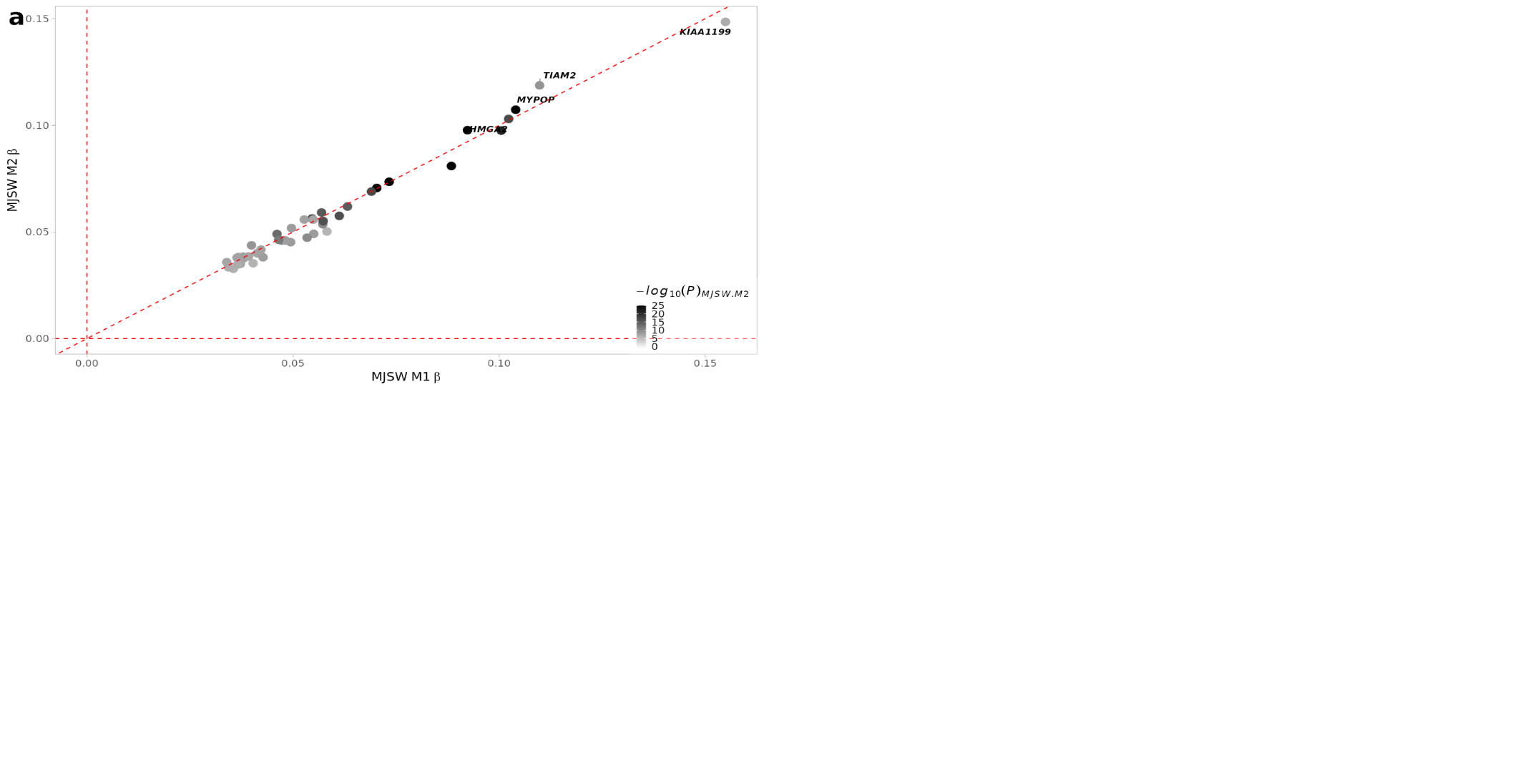


## Supplementary figure 3: Look up of a) Model 1 (adjusted for age and sex) mJSW meta-analysis results in Model 2 mJSW meta analysis results (adjusted for height) b) Look up of Model 2 results in Model 1

a b c


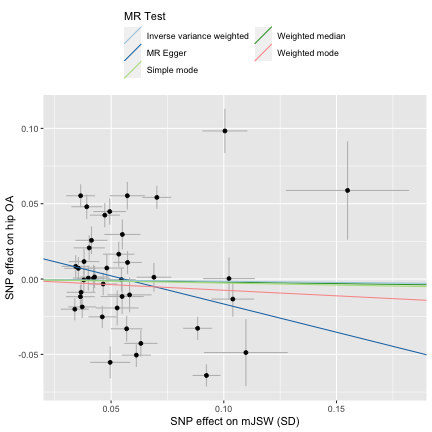

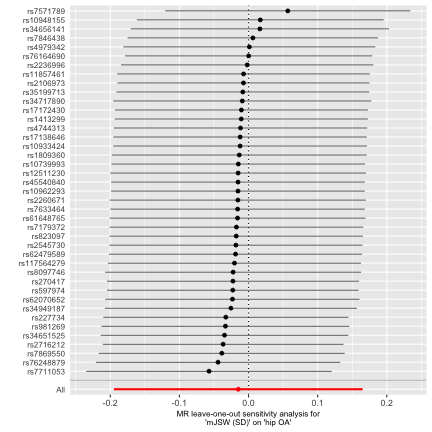

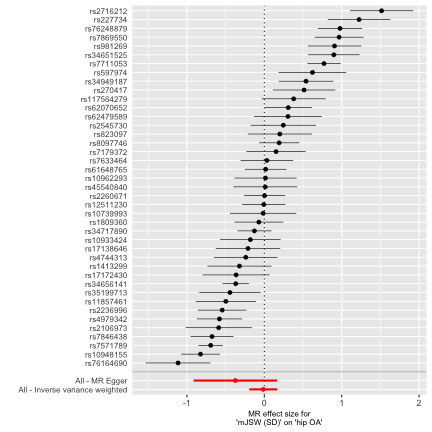


## Supplementary Figure 4. Exposure – mJSW, Outcome – hip osteoarthritis. a – Mendelian randomisation plot comparing the 5 different methods. b – leave one out analysis. c – Single SNP analysis.


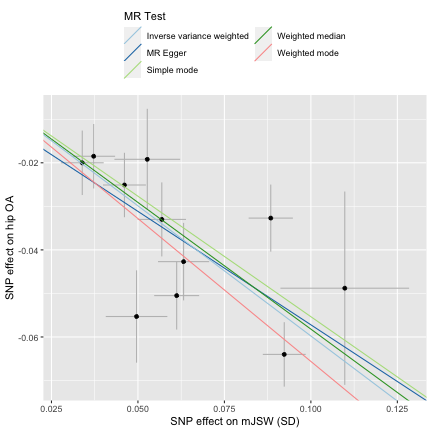

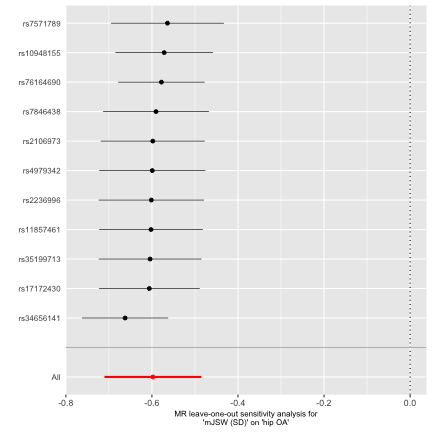

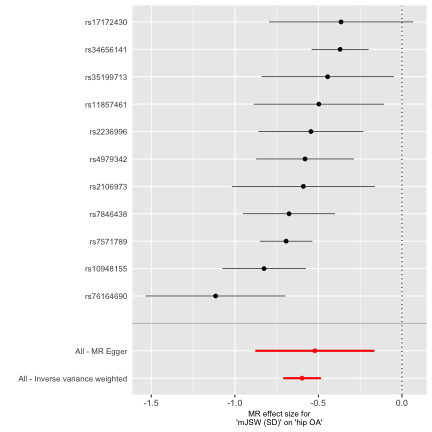


a b c

## Supplementary Figure 5. Exposure – Cluster one mJSW SNPs, Outcome – hip osteoarthritis. a – Mendelian randomisation plot comparing the 5 different methods. b – leave one out analysis. c – Single SNP analysis.


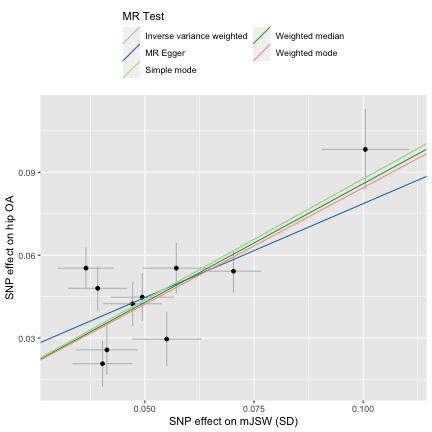

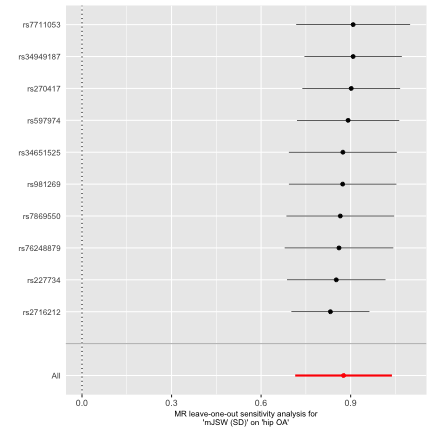

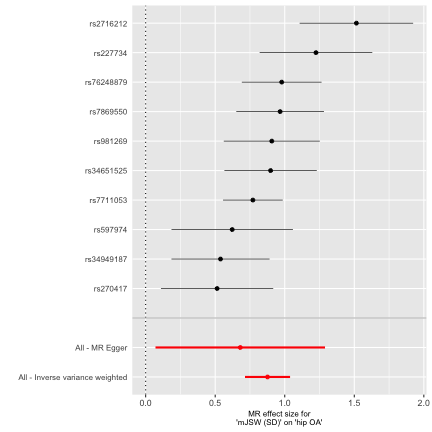


a b c

## Supplementary Figure 6. Exposure – Cluster two mJSW SNPs, Outcome – hip osteoarthritis. a – Mendelian randomisation plot comparing the 5 different methods. b – leave one out analysis. c – Single SNP analysis.


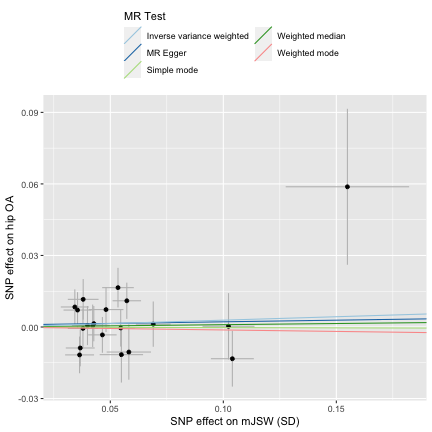

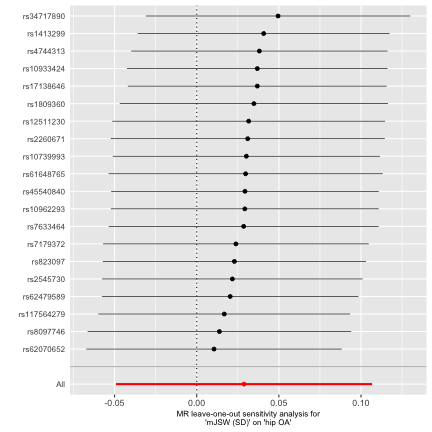

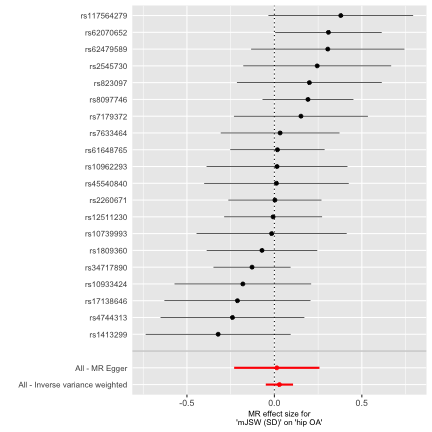


a b c

## Supplementary Figure 7. Exposure –Cluster three mJSW SNPs, Outcome – hip osteoarthritis. a – Mendelian randomisation plot comparing the 5 different methods. b – leave one out analysis. c – Single SNP analysis.


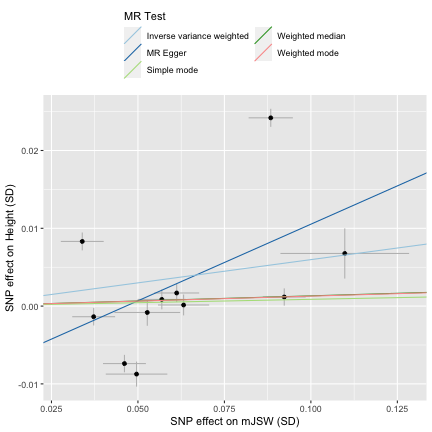

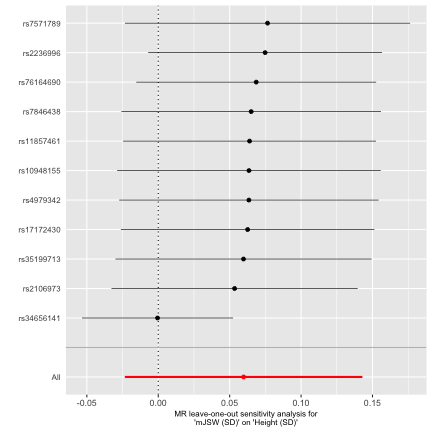

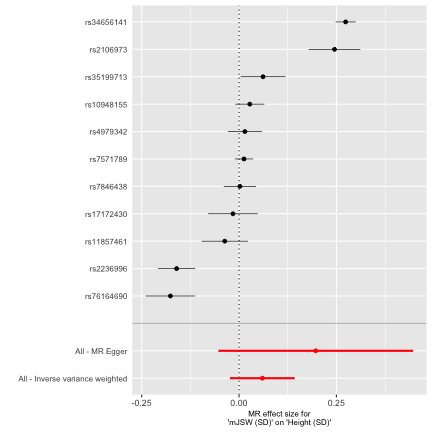


a b c

## Supplementary Figure 8. Exposure – Cluster one mJSW SNPs, Outcome – height. a – Mendelian randomisation plot comparing the 5 different methods. b – leave one out analysis. c – Single SNP analysis.


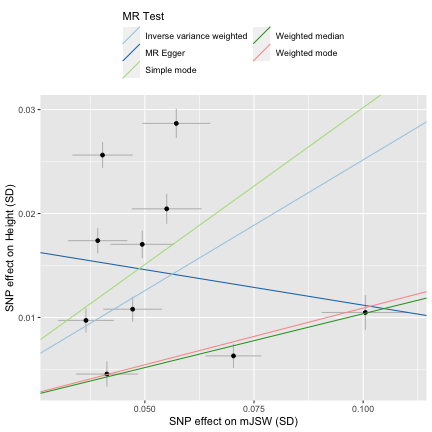

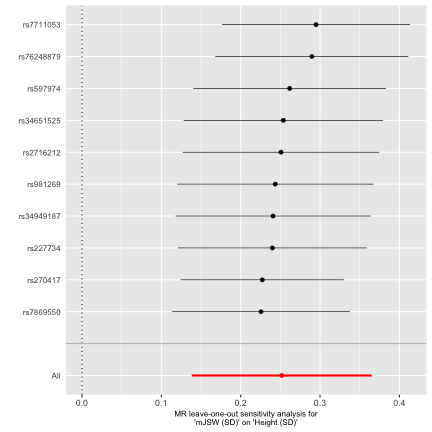

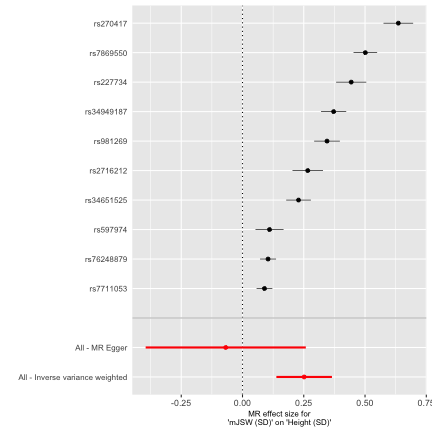


a b c

## Supplementary Figure 9. Exposure – Cluster two mJSW SNPs, Outcome – height. a – Mendelian randomisation plot comparing the 5 different methods. b – leave one out analysis. c – Single SNP analysis.
